# Accelerating and Enhancing Thermodynamic Simulations of Electrochemical Interfaces

**DOI:** 10.1021/acscentsci.5c00547

**Published:** 2025-07-21

**Authors:** Xiaochen Du, Mengren Liu, Jiayu Peng, Hoje Chun, Alexander Hoffman, Bilge Yildiz, Lin Li, Martin Z. Bazant, Rafael Gómez-Bombarelli

**Affiliations:** † Department of Chemical Engineering, 2167 Massachusetts Institute of Technology, Cambridge, Massachusetts 02139, United States; ‡ Department of Materials Science and Engineering, 2167 Massachusetts Institute of Technology, Cambridge, Massachusetts 02319, United States; ¶ Department of Nuclear Science and Engineering, 2167 Massachusetts Institute of Technology, Cambridge, Massachusetts 02319, United States; § Massachusetts Institute of Technology Lincoln Laboratory, Lexington, Massachusetts 02421, United States

## Abstract

Electrochemical interfaces are crucial in catalysis, energy storage, and corrosion, where their stability and reactivity depend on complex interactions between the electrode, adsorbates, and electrolyte. Predicting stable surface structures remains challenging, as traditional surface Pourbaix diagrams tend to either rely on expert knowledge or costly *ab initio* sampling and neglect thermodynamic equilibration with the environment. Machine learning (ML) potentials can accelerate static modeling but often overlook dynamic surface transformations. Here, we extend the Virtual Surface Site Relaxation-Monte Carlo (VSSR-MC) method to autonomously sample surface reconstructions modeled under aqueous electrochemical conditions. Through fine-tuning foundational ML force fields, we accurately and efficiently predict surface energetics, recovering known Pt(111) phases and revealing new LaMnO_3_(001) surface reconstructions. By explicitly accounting for bulk-electrolyte equilibria, our framework enhances electrochemical stability predictions, offering a scalable approach to understanding and designing materials for electrochemical applications.

## Graphical abstract

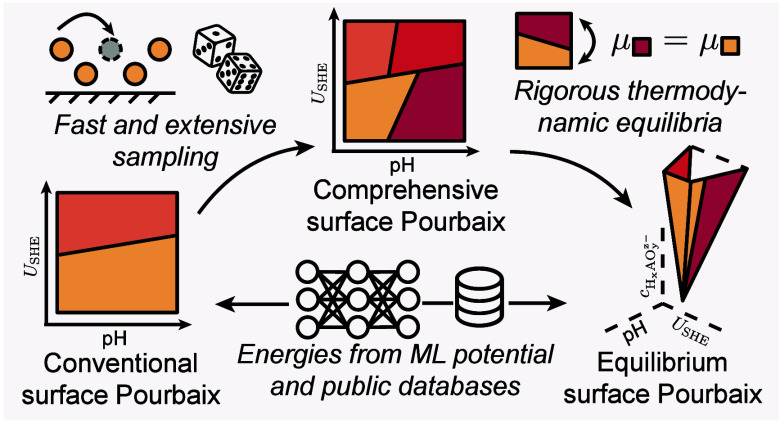

## Introduction

The structure of surfaces and interfaces critically influences the performance and stability of materials in applications ranging from (electro-)­catalysis^1–3^ and energy storage^4–6^ to electronic devices.^7,8^ In gas or vacuum environments, a nominally pristine surface derived by cleaving the material bulk often undergoes atomic rearrangements that can produce intricate reconstructions, as documented for materials such as Si,^9–11^ GaN,^12,13^ and α-Al_2_O_3_.^14^ Under aqueous electrochemical conditions, other factors influence the stability of the surface: the electrolyte pH, the applied electrical potential, the presence of adsorbates such as OH* and H_2_O*,^15–24^ and the formation of charged layers.^25–27^ These added variables drive the dissolution and redeposition of metal surface atoms, posing significant challenges for understanding and predicting the structure and stability of electrochemical interfaces.^28–30^

Pourbaix diagrams, which delineate thermodynamically favored phases as a function of pH and electrical potential, are a cornerstone in assessing electrochemical material stability. An individual species Pourbaix diagram shows the most stable bulk or dissolved species of an element, whereas a surface Pourbaix diagram illustrates the most stable surface structure given a fixed bulk phase. Both types of Pourbaix diagrams have guided the design of corrosion prevention strategies,^31,32^ informed the development of electrocatalysts,^30,33–38^ and aided in planning rational synthesis routes.^39,40^

In recent decades, first-principles computational methods have enhanced the ability to construct surface Pourbaix diagrams by predicting atomic-level interfacial energetics.^33,39,41,42^ However, many such studies were limited to pristine facets with predefined adsorbate coverages, neglecting more complex reconstructions or adsorption patterns under operational conditions.^33,42–47^ Moreover, traditional Pourbaix diagrams often assume a fixed concentration for dissolved species (commonly 10^–5^ to 10^–6^ M).^39,40,48–53^ While computationally convenient, this assumption disregards true equilibration between the bulk phase and its dissolved ions. As a result, important stability behaviors may be obscured, especially when designing long-lifetime catalysts based on bulk-electrolyte equilibration principles^54,55^ or synthesizing materials that are only stable at higher dissolved-ion concentrations.^40^

Studies considering surface dissolution, redeposition, or interchanges within multicomponent systems often rely on “hand-crafted” structural models validated by costly first-principles calculations, a method that becomes insufficient for exploring complex multicomponent systems with large compositional and configurational spaces.^16,24,49,56^ Recent efforts to sample across the compositions of a binary system nevertheless relied on costly *ab initio* grand canonical Monte Carlo (GCMC) sampling that might not scale to more complex materials.^57^

Machine learning (ML) techniques, especially neural network force fields (NFFs), offer a pathway to address these challenges by enabling faster atomistic modeling,^58–65^ but current approaches overlook complex surface reconstruction phenomena. The growing availability of pretrained, foundational NFFs expands the applicability of these methods to new material classes with limited density-functional theory (DFT) data.^66–71^ However, most ML-based approaches in catalysis either focus on high-throughput screening of ideal surfaces with predefined adsorbate patterns^65,72–77^ or examine reconstruction only within a narrow set of configurations—permitting select adsorbate and metal surface combinations but disallowing general surface dissolution and redeposition—thus leaving the broader compositional space largely unexplored.^53,78–82^

Adapting thermodynamic sampling approaches developed for gas- or vacuum-exposed surfaces, such as the Virtual Surface Site Relaxation-Monte Carlo (VSSR-MC) algorithm,^83^ can capture more realistic reconstruction phenomena. Extending such approaches to electrochemical conditions requires accounting for pH, applied electrical potential, and relevant dissolved species concentrations, along with a robust treatment of the complex equilibria between the electrolyte, the electrode surface, and the electrode bulk.

In this work, we develop a comprehensive methodology that addresses these challenges in three ways. First, we adapt the VSSR-MC sampling approach to electrochemical conditions, enabling automated discovery of stable surface reconstructions across compositional and configurational spaces involving both adsorbates and underlying metal species. Second, we demonstrate how pretrained NFFs can be fine-tuned to achieve DFT-level accuracy for surface energetics while maintaining computational efficiency. We also assess when such fine-tuning is necessary by comparing cases where pretrained models perform well versus fall short. Third, we establish a theoretical framework for calculating surface Pourbaix diagrams that explicitly accounts for dynamic equilibria with the electrode bulk and aqueous species at varying concentrations.

We validate our approach through two case studies of increasing complexity. Using Pt(111) as a benchmark, we demonstrate the ability to efficiently sample known surface reconstructions and show that fine-tuning is essential for recovering DFT-consistent thermodynamics. We then tackle the more challenging case of LaMnO_3_(001), where the pretrained model already performs well, but additional sampling reveals new stable surface phases. Finally, we highlight the importance of capturing electrolyte-bulk equilibria by constructing surface Pourbaix diagrams that respect the thermodynamic coupling between electrolyte species, the electrode surface, and the electrode bulk. These case studies showcase the ability of our method to predict realistic electrochemical interfaces, accelerating the discovery of stable surfaces for energy conversion and catalysis.

## Results

### Computational Workflow and Method Development

The original VSSR-MC method was developed in ref 83 to enable efficient sampling of surface reconstructions under vacuum or gas-phase conditions. Briefly, VSSR-MC performs Markov-chain Monte Carlo (MCMC) sampling in the semigrand canonical ensemble using a set of algorithmically generated virtual surface sites defined above the pristine surface. These sites discretize the surface configurational space and can host atoms or remain empty, allowing compositional changes to be represented as identity swaps at a single site (with “empty” treated as a valid identity) rather than as explicit positional insertions. This approach avoids the high-dimensional continuous space of off-lattice GCMC while efficiently capturing relevant reconstructions. The improvement of VSSR-MC over prior state-of-the-art methods is documented in the original work (Supplementary Data 1).^83^

Each VSSR-MC iteration involves four key steps: (1) enumeration of possible discrete changes at a selected site, (2) application of the chosen change to generate a new composition, (3) local continuous relaxation of atomic positions, and (4) Metropolis-Hastings acceptance based on the semigrand ensemble. Sterically unfavorable configurations are rejected via hard-sphere cutoffs, and continuous relaxation enables exploration of low-energy configurations. Importantly, VSSR-MC is agnostic to the energy model—it supports classical potentials or NFFs trained via active learning to reproduce any chosen level of DFT, including specific exchange-correlation functionals.

In this work, we extend VSSR-MC beyond its original formulation^83^ to sample multiatom adsorbates such as OH* and H_2_O*, in addition to single-atom adsorbates such as O* and metal species, at the virtual sites (see “Surface Slab Modeling” in the Methods section). Additionally, we included the pristine surface atoms as virtual sites (see Figures S2­(b) and S5­(b)) to simulate dissolution and redeposition, thereby broadening the search space of possible reconstructions.

To evaluate each Monte Carlo (MC) move under electrochemical conditions, the energy difference between the proposed and current slab is calculated via a two-step process to account for the dominant species of each element under aqueous electrochemical conditions.^49,56^ In Figure 1(a), we consider the case of a single atom dissolution; for more complex MC steps or to compare stability between two arbitrary surfaces, a sequence of dissolution/adsorption steps is constructed to calculate the total energy change. In step 1, atom A is removed from the surface—creating a vacancy—and released in its standard state, yielding energy change ΔΩ_1_. In step 2, the freed atom converts to the most stable aqueous species with general formula H_*x*_AO_*y*_^*z* ^–^^, following the computational hydrogen electrode (CHE) framework,^84^ with energy change ΔΩ_2_. The specific H_*x*_AO_*y*_^*z* ^–^^ identity depends on thermodynamic conditions (see “Species Pourbaix Diagram Construction” and “Surface Pourbaix Grand Potential” in the Methods section). The overall energy difference is given by Eqn. [Disp-formula eq1]:

1 $$\Delta \Omega_{surf}\left(X,\left\{\mu \right\},T,U_{SHE},pH\right)=\Delta \Omega_{1}+\Delta \Omega_{2}$$

where **X** refers to the atomic identities and positions, {μ} represents the set of chemical potentials of the stable aqueous species $\mu_{H_{x}{AO}_{y}^{z-}}$ (related to species concentration), *T* is the temperature, and *U*_SHE_ is the electrical potential relative to the standard hydrogen electrode (SHE). The surface Pourbaix grand potential, Ω_surf_(*U*_SHE_, pH), of each slab can thus be interpreted as the surface formation energy derived from the stable aqueous species at a given *U*_SHE_ and pH (with fixed aqueous species concentrations and temperature). See “Surface Pourbaix Grand Potential” in the Methods section for details.

**Figure 1:**
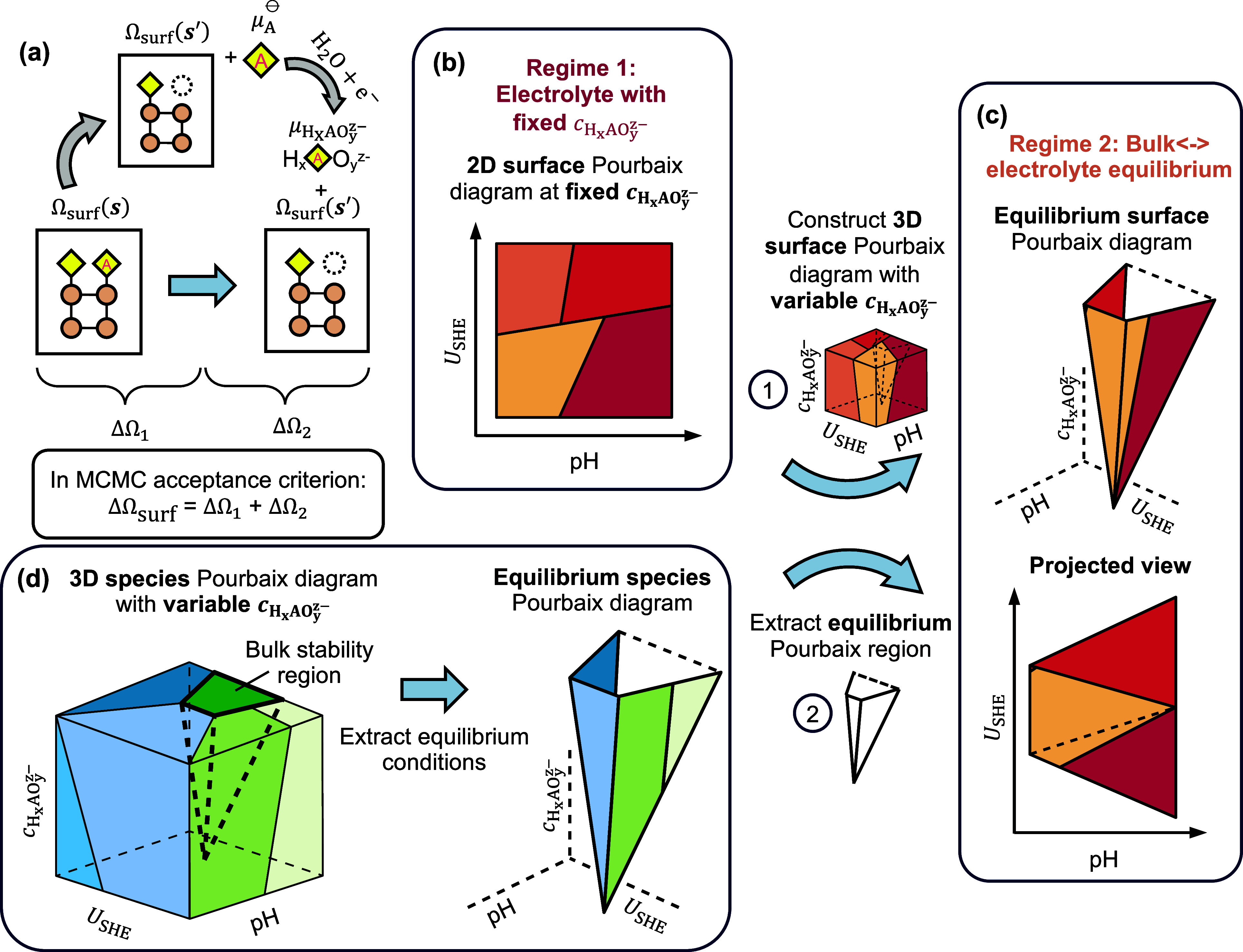
**Thermodynamic analysis for electrochemical interfaces.** (a) Two-step process for comparing surface Pourbaix grand potentials (Ω_surf_(*U*_SHE_, pH)) between two slabs. The figure shows the case of a single-atom dissolution, but more complicated changes can be constructed as a sequence of dissolution/adsorption steps. (b, c) Two regimes for constructing surface Pourbaix diagrams. (b) Conventional surface Pourbaix diagram at fixed dissolved species concentrations. (c) Equilibrium surface Pourbaix diagram derived from bulk-electrolyte equilibrium conditions. (d) To construct the equilibrium surface Pourbaix diagram, the 3D species Pourbaix diagram is generated to define the equilibrium conditions for bulk stability in pH-*U*_SHE_- $c_{H_{x}{AO}_{y}^{z-}}$ space. Afterward, a 3D surface Pourbaix diagram is constructed from the surface Pourbaix diagram in (b) and the equilibrium Pourbaix region traced out to produce the final equilibrium surface Pourbaix diagram.

We construct surface Pourbaix diagrams under two distinct thermodynamic regimes. In regime 1 (Figure 1(b)), the conventional surface Pourbaix diagram is plotted at a fixed dissolved species concentration (*c*_H_*x*_AO_*y*_^*z*–^_) in the electrolyte, which acts as the thermodynamic reservoir. Here, stable aqueous species for each metal atom are identified at each pH and *U*_SHE_ using the species Pourbaix diagram. Since each domain in the species Pourbaix diagram corresponds to a distinct set of dominant species, we compute separate Ω_surf_(*U*_SHE_, pH) values for all considered surface slabs within each domain. A convex hull analysis of these grand potentials reveals the stable surface domains,^48,50,52,85^ which may be the same across species domain boundaries. Thus, all surface domains are pooled and merged to produce the final surface Pourbaix diagram. A detailed schematic is provided in Figure S1.

In regime 2 (Figure 1(c)), we introduce a bulk-electrolyte equilibrium analysis where both the electrode bulk and the electrolyte serve as thermodynamic reservoirs in equilibrium with the surface. In this regime, we plot the equilibrium surface Pourbaix diagram by extending the conventional Pourbaix diagram into three dimensions to explicitly incorporate $c_{H_{x}{AO}_{y}^{z-}}$ as a variable dependent on pH and *U*_SHE_. First, a three-dimensional (3D) species Pourbaix diagram is generated with an additional, independent $c_{H_{x}{AO}_{y}^{z-}}$ axis. By following the phase boundaries of the bulk stability region in pH-*U*_SHE_- $c_{H_{x}{AO}_{y}^{z-}}$ space, we trace out the equilibrium species Pourbaix diagram that captures the range of conditions under which the electrode bulk remains stable and in equilibrium with the neighboring electrolyte species. In this equilibrium Pourbaix diagram, pH, *U*_SHE_, and $c_{H_{x}{AO}_{y}^{z-}}$ are thermodynamically coupled rather than independent variables. See Figure 1(d) for an illustration and “Thermodynamic Equilibria” in the Methods section for details. The equilibrium surface Pourbaix diagram is constructed analogously by generating a 3D surface Pourbaix diagram (using a workflow similar to Figure S1) and delineating the same bulk stability boundaries (details also in “Thermodynamic Equilibria” in the Methods section). Projecting the 3D equilibrium surface Pourbaix diagram into the pH-*U*_SHE_ plane yields a diagram that resembles a conventional Pourbaix diagram but with $c_{H_{x}{AO}_{y}^{z-}}$ as a dependent variable.

### Assessing Pretrained and Fine-Tuned NFF Performance

To assess when fine-tuning NFFs is necessary for surface Pourbaix diagram construction, we compared two representative systems: Pt(111), a canonical metal electrocatalyst surface with localized adsorbates,^3,86^ and LaMnO_3_(001), a perovskite oxide with chemically diverse environments relevant for both oxygen-evolution (OER) and oxygen-reduction (ORR) reactions.^49,87^ Surfaces were sampled using our modified VSSR-MC approach and evaluated using both pretrained NFFs and models fine-tuned on a subset of samples. These comparisons highlight how the chemical proximity of surface structures to bulk materials in the pretraining data set impacts the need for fine-tuning.

### Validation on a Metal Surface

We first evaluated 8 Pt(111) surfaces from the literature featuring various O* and stoichiometric [OH–H_2_O]* coverages^33,42^ (see “Surface Slab Modeling” in the Methods section). We used CHGNet^67^ as our pretrained NFF model, as its training set already incorporates energy corrections from the Materials Project 2020 compatibility scheme,^88^ which we also used to align energies for Ω_surf_(*U*_SHE_, pH).^48,50,85^ Using pretrained CHGNet, relative surface Pourbaix grand potentials (ΔΩ_surf_(*U*_SHE_, pH)) with respect to the pristine surface at fixed pH = 0 and varying *U*_SHE_ followed the correct trend across O* levels (see “Surface Nomenclature” in the SI for our slab-naming convention), but were significantly overestimated compared to DFT (Figure 2(a,b)). The predicted surface Pourbaix diagram (at 10^–6^ M dissolved species concentration) was also shifted and mispredicted one adlayer phase relative to DFT (Figure 2(d,e)). CHGNet overpredicts these grand potentials because it was pretrained on the MPtrj data set—which consists only of bulk crystals^89^—and thus does not generalize well to Pt surfaces with O* and OH* adsorbates.

**Figure 2:**
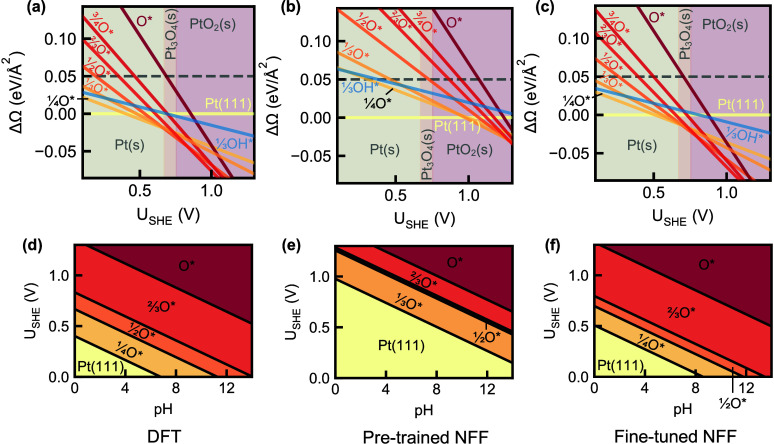
**Pt­(111) energy analysis and surface Pourbaix diagrams with various energy models.** (a–c) ΔΩ_surf_(*U*_SHE_, pH) comparison of handpicked Pt(111) surfaces with respect to the pristine surface in eV/Å^2^ across *U*_SHE_ at fixed pH = 0 with (a) DFT, (b) pretrained NFF (CHGNet), and (c) fine-tuned NFF (MACE) energies. The bulk species domains are differentiated through the background color and labeled in gray. Dashed lines are a guide for the eye. (d–f) Pt(111) surface Pourbaix diagrams for (d) DFT, (e) pretrained NFF, and (f) fine-tuned NFF.

VSSR-MC sampling reproduced all handpicked surfaces from the literature (example profile in Figure S3; see “VSSR-MC” in the Methods section for details). Fine-tuning on 155 representative structures (see “Active Learning” in the Methods section), including both sampled and rattled configurations, substantially reduced errors and corrected phase ordering (Figure 2(c,f)). We fine-tuned both CHGNet and MACE,^68,90^ and found that MACE performed significantly better despite being pretrained on uncorrected DFT energies (Figure S4). Fine-tuned MACE achieved ΔΩ_surf_(*U*_SHE_, pH) errors within 10 meV/Å^2^, sufficient to reproduce DFT-level surface stability distinctions.

### Validation on an Oxide Surface

By contrast, evaluating 47 handpicked 2 × 2 LaMnO_3_(001) surfaces with varying levels of metal vacancies and O*/OH* coverages^49^ using the same pretrained CHGNet yielded surface Pourbaix diagrams closely aligned with the DFT reference (Figure 3(a,b)), recovering most dominant phases and capturing qualitative oxidation trends. (See “Surface Slab Modeling” in the Methods section for modeling details and “Surface Nomenclature” in the SI for our slab-naming convention.) The pretrained CHGNet energy mean absolute error (MAE) was ∼0.1 eV/atom—almost an order of magnitude better than for Pt(111) (Figure S7­(a) vs Figure S4­(a)). We attribute this to the similarity between LaMnO_3_(001) surface chemistry and the CHGNet training set, which includes oxide-rich materials.

**Figure 3:**
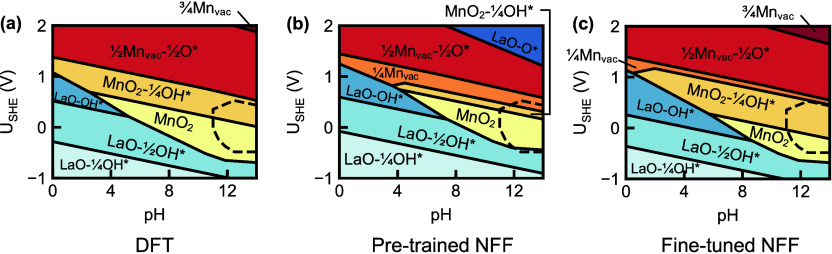
**LaMnO**_**3**_ **(001) surface Pourbaix diagrams with various energy models.** LaMnO_3_(001) surface Pourbaix diagrams from energies computed with (a) DFT, (b) pretrained NFF, and (c) fine-tuned NFF. The LaMnO_3_ bulk stability regions at 10^–6^ M dissolved species concentrations are enclosed by the dashed lines.

VSSR-MC sampling was also performed for LaMnO_3_(001) (example profile in Figure S6; see “VSSR-MC” in the Methods section), focusing on the bulk stability region. Fine-tuning CHGNet on 136 VSSR-MC and rattled structures (see “Active Learning” in the Methods section) led to modest improvements: refined phase boundaries (Figure 3(c)) and slightly more accurate energetics (Figures S7­(b) and S8­(c)). See “Additional LaMnO_3_(001) Surface Pourbaix Analysis” in the SI for details.

These results suggest that pretrained NFFs can generalize well to complex oxide surfaces with familiar bonding motifs, but fine-tuning remains crucial for systems outside the training distribution, such as Pt with sparse O*/OH* adlayers. In practice, even limited fine-tuning enables reliable Pourbaix predictions at a fraction of the cost of full DFT sampling or training a model from scratch.^57,83,91^

### Sampling Additional Surface Phases

Apart from sampling known reconstructions, our study of LaMnO_3_(001) uncovered surfaces not identified in previous work. These newly sampled structures are represented as gray lines in the relative surface Pourbaix grand potential plot in Figure 4(a) using fine-tuned CHGNet energies at pH = 12 (see “Surface Analysis” in the Methods section for details). Some of these additional surfaces are predicted to be even more stable than the low-energy handpicked structures. Subsequent DFT energy evaluations confirm their stability, leading to a revised LaMnO_3_(001) surface Pourbaix diagram in Figure 4(b). In contrast, the sampled structures for Pt(111) were not found to be lower in energy than reference terminations, suggesting that the set of reference terminations is already reasonably complete within our sampling framework. Although our sampling did not yield lower-energy structures beyond reference terminations, recent work has shown that Pt(111) can undergo further surface reconstructions under electrochemical conditions, in which a surface Pt atom is displaced from its lattice site and the resulting vacancy is often filled by an oxygen atom.^92^ Capturing such reconstructions may require additional VSSR-MC sampling using fine-tuned force fields. As a result, Pt(111) surfaces were not further analyzed. (See Figure S9 for the equivalent Pt(111) surface Pourbaix grand potential plot.)

**Figure 4:**
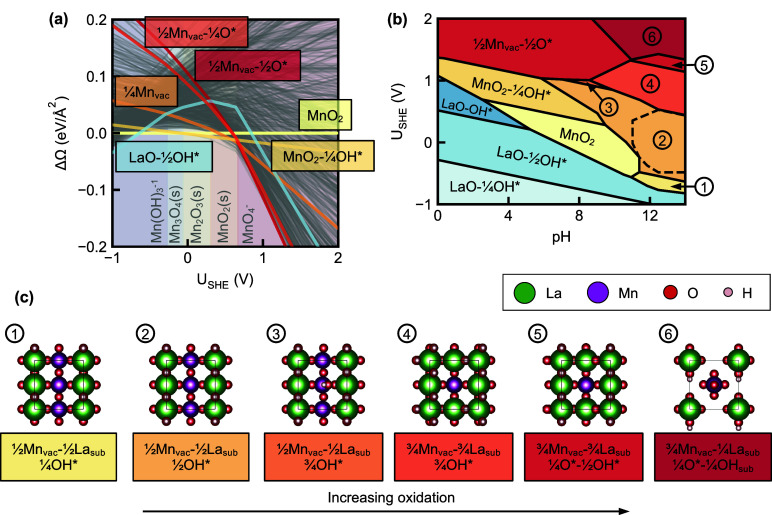
**LaMnO**_**3**_ **(001) energy analysis and surface Pourbaix diagrams.** (a) ΔΩ_surf_(*U*_SHE_, pH) with respect to the pristine surface in eV/Å^2^ of handpicked and VSSR-MC sampled structures evaluated with fine-tuned CHGNet energies across *U*_SHE_ at fixed pH = 12. Handpicked structures are labeled, while sampled structures are grayed. (b) Revised LaMnO_3_(001) surface Pourbaix diagram at the DFT level with additional stable phases sampled using VSSR-MC. (c) Top-down view of stable sampled phases colored approximately in increasing oxidation level.

The newly identified phases for LaMnO_3_(001) (Figure 4(c)) demonstrate complex surface chemistry, featuring mixed La and Mn terminations that were absent from the handpicked structures but have been experimentally observed in LaMnO_3_(001) reconstructions under ORR settings.^93^ In ref 93, enhanced ORR activity in LaMnO_3_ was attributed to the coexistence of Mn^2+^ and Mn^3+^ species at the surface layers. This mixed Mn oxidation state arises from the La and Mn mixed-termination that is distinct from the bulk structure. Among the VSSR-MC sampled structures, we identified a total of 23 such mixed-termination surfaces (Figure S10) more stable than the most stable surface among the handpicked structures (MnO_2_- $\frac{1}{4}$ OH*) at pH = 12 and *U*_SHE_ = 0.6 V.

The stability trend of the new LaMnO_3_(001) surface Pourbaix diagram (Figure 4(b)) with respect to electrical potential is consistent with our analysis of Figure 3(a) (see “Additional LaMnO_3_(001) Surface Pourbaix Analysis” in the SI). At the most negative *U*_SHE_ at pH = 12, the most stable surface composition features a LaO termination with $\frac{1}{2}$ OH*. Increasing *U*_SHE_ induces progressive oxidation, first by introducing Mn to form a mixed-termination structure, followed by a reduction of Mn occupancy in the termination layer. At the same time, more oxidizing species appear on the adlayer, first with OH* adsorption, followed by O* adsorption.

On top of sampling 2 × 2 surfaces, we performed additional sampling for 3 × 3 and 4 × 4 LaMnO_3_(001) using fine-tuned CHGNet. The energetics of these structures are illustrated in the relative surface Pourbaix grand potential plots at pH = 12 (Figure S11­(b,c)), which have a higher density at lower ΔΩ_surf_(*U*_SHE_, pH) compared with those of the 2 × 2 supercell (Figure 4(a), reproduced in Figure S11­(a)). Using fine-tuned CHGNet energies, a total of 88 structures for the 3 × 3 and 33 structures for the 4 × 4 supercells were predicted to be at most 0.1 eV/surface unit cell (6.42 meV/Å^2^) higher in energy than the initially most stable surface, MnO_2_- $\frac{1}{4}$ OH*, at pH = 12 and *U*_SHE_ = 0.6 V. This is more than the 27 structures predicted to be in the same energy range for the 2 × 2 supercell. We constructed individual surface Pourbaix diagrams at each supercell size using fine-tuned CHGNet as the energy model (Figure S11­(d–f)). While the 2 × 2 supercell yielded only 3 additional phases at the fine-tuned CHGNet level, the 3 × 3 supercell displayed 7 additional phases. The 4 × 4 supercell also revealed 3 additional phases. All these stable surfaces are visualized in Figure S12. Finally, we combined handpicked structures with those sampled across all three supercell sizes to produce a unified surface Pourbaix diagram (Figure S11­(g)). Among the 5 new surface Pourbaix domains identified, 2 originated from a larger supercell. These structures were not evaluated using DFT but nevertheless show that VSSR-MC is capable of interfacing with larger symmetry-breaking supercells to uncover additional surface phases.

In summary, VSSR-MC sampled across compositions and configurations to discover over 3500 unique LaMnO_3_(001) surface structures, far greater than the 47 handpicked surfaces initially considered, of which 6 structures were verified with DFT to be more stable than the handpicked structures.

### New Surface-Environment Equilibria Conditions

After benchmarking our sampling method and NFF fine-tuning with conventional Pourbaix diagrams, we re-examine the thermodynamic assumptions and replot Pourbaix diagrams under bulk-electrolyte equilibrium conditions. We assumed a 10^–6^ M concentration for the stable dissolved species at each pH and *U*_SHE_ to generate the previous LaMnO_3_(001) surface Pourbaix diagrams in keeping with literature convention. Here, we consider surface Pourbaix diagrams where $c_{H_{x}{AO}_{y}^{z-}}$ is thermodynamically coupled to pH and *U*_SHE_ to account for equilibrium between the LaMnO_3_ bulk and metal species in the electrolyte. These equilibrium surface Pourbaix diagrams are a subset of the full 3D surface Pourbaix diagrams in pH-*U*_SHE_- $c_{H_{x}{AO}_{y}^{z-}}$ space where $c_{H_{x}{AO}_{y}^{z-}}$ varies as an independent variable alongside pH and *U*_SHE_.

Meanwhile, 3D surface Pourbaix diagrams were generated from the 3D LaMnO_3_ species Pourbaix diagram. Hence, as a first step toward generating the 3D surface Pourbaix diagrams, we extend the conventional LaMnO_3_ species Pourbaix diagram into 3D (Figure S14), where the dissolved species concentration varies as an independent variable alongside pH and *U*_SHE_, to compute thermodynamic equilibration between the electrode bulk and electrolyte species in the pH-*U*_SHE_- $c_{H_{x}{AO}_{y}^{z-}}$ space. The resultant equilibrium species Pourbaix diagrams (Figure 5(a,b)) show the LaMnO_3_ bulk-electrolyte species equilibrium Pourbaix region delineated from the 3D species Pourbaix diagram. (See “Thermodynamic Equilibria” in the Methods section for details.) The domains are labeled according to the species adjacent to the bulk stability region in the 3D species Pourbaix diagram, which represent species present in the electrolyte. Figure 5(a) shows the 3D perspective while Figure 5(b) shows a projected view in the pH-*U*_SHE_ axes, with $c_{H_{x}{AO}_{y}^{z-}}$ implicitly defined by thermodynamic equilibrium. The pH and *U*_SHE_ ranges spanned roughly correspond to the ORR region bounded by – 0.709 eV < *U*_SHE_ < 0.521 eV at pH = 12, and are therefore much narrower than those in a conventional Pourbaix diagram. As we decrease $c_{H_{x}{AO}_{y}^{z-}}$ from 10^–3^ M to 10^–15^ M, the stability region further narrows, aligning with Le Chatelier’s principle on chemical equilibrium between the LaMnO_3_ bulk and adjacent species. From another perspective, any set of pH and *U*_SHE_ conditions within the bulk stability region corresponds to a predefined $c_{H_{x}{AO}_{y}^{z-}}$ . In principle, we can indefinitely extend the bulk stability region by increasing $c_{H_{x}{AO}_{y}^{z-}}$ . In practice, however, $c_{H_{x}{AO}_{y}^{z-}}$ is limited by species solubility and device durability considerations.^54^

**Figure 5:**
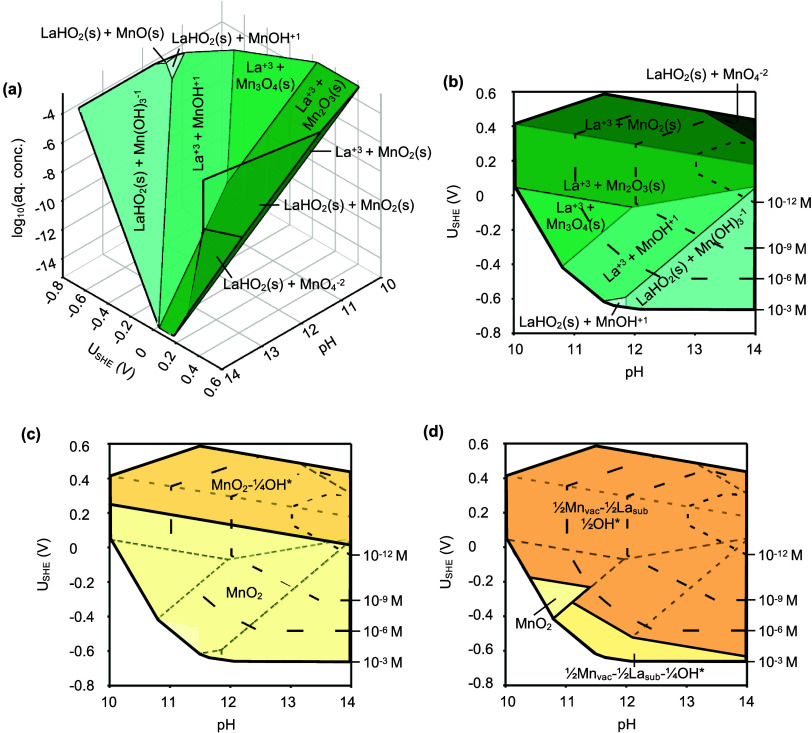
**LaMnO**_**3**_ **species and LaMnO**_**3**_ **(001) surface Pourbaix diagrams at thermodynamic equilibrium in the pH-** ***U***_**SHE**_ **-** $\log_{10}c_{H_{x}{AO}_{y}^{z-}}$ **axes.** Phases are colored approximately in increasing oxidation level from pale to dark. The black dashed contour lines correspond to different $c_{H_{x}{AO}_{y}^{z-}}$ . (a, b) Equilibrium species Pourbaix diagrams labeled by species domains adjacent to the bulk stability region. (a) 3D perspective. (b) 2D projection in the pH-*U*_SHE_ axes from high $c_{H_{x}{AO}_{y}^{z-}}$ . (c, d) 2D projections of equilibrium surface Pourbaix diagrams with (c) only literature surfaces and (d) including additional sampled surfaces.

Outside of the bulk stability region, LaMnO_3_ preferentially transforms into other La- and Mn-containing phases or dissolves entirely. In such cases, the assumption of a stable LaMnO_3_ bulk supporting the surface is no longer valid, as the underlying bulk structure itself changes. To ensure a meaningful surface Pourbaix analysis, we must therefore account for surface equilibration with both metal species in the electrolyte as well as the LaMnO_3_ bulk. We extend the conventional surface Pourbaix diagrams into 3D surface Pourbaix diagrams with an additional, independent $c_{H_{x}{AO}_{y}^{z-}}$ axis in Figures S15 and S16, and extract the same equilibrium Pourbaix region to respectively generate the equilibrium surface Pourbaix diagram with literature reported surfaces in Figure 5(c) and the equilibrium surface Pourbaix diagram with both literature and additional sampled structures in Figure 5(d). Like Figure 5(b), these plots are 2D projections that resemble conventional Pourbaix diagrams across pH-*U*_SHE_ axes but with $c_{H_{x}{AO}_{y}^{z-}}$ implicitly defined. Full 3D views in Figure S17 also illustrate this thermodynamic coupling between pH, *U*_SHE_, and $c_{H_{x}{AO}_{y}^{z-}}$ . The pH and *U*_SHE_ ranges are constrained by the equilibrium species Pourbaix diagram in Figure 5(a,b), resulting in a narrower range compared to a conventional surface Pourbaix diagram, which shares limits with the conventional species Pourbaix diagram. Consequently, Figure 5(c,d) show fewer phases than Figures S15 and S16. Figure 5(c) contains exactly two phases—pristine MnO_2_ and MnO_2_- $\frac{1}{4}$ OH*. With the inclusion of sampled surfaces in Figure 5(d), the MnO_2_- $\frac{1}{4}$ OH* phase disappears and the MnO_2_ stability region shrinks. Most of the surface Pourbaix diagram is now covered by two newly identified surfaces in Figure 4(c), with $\frac{1}{2}$ Mn_vac_- $\frac{1}{2}$ La_sub_- $\frac{1}{4}$ OH* occupying an elongated region at the bottom while $\frac{1}{2}$ Mn_vac_- $\frac{1}{2}$ La_sub_- $\frac{1}{2}$ OH* dominating the remainder. Other sampled surfaces from Figure 4(c) do not appear here as they fall outside of the bulk stability region. The slightly warped phase boundary of the $\frac{1}{2}$ Mn_vac_- $\frac{1}{2}$ La_sub_- $\frac{1}{4}$ OH* phase arises due to variations in $c_{H_{x}{AO}_{y}^{z-}}$ .

In an aqueous electrochemical system where the electrode equilibrates with the electrolyte, the equilibrium surface Pourbaix diagram illustrates how the dominant surface phase evolves as a function of pH, *U*_SHE_, and $c_{H_{x}{AO}_{y}^{z-}}$ . Any two of these three variables uniquely determine the third; for instance, fixing $c_{H_{x}{AO}_{y}^{z-}}$ and *U*_SHE_ allows us to determine the equilibrium pH in addition to the predominant surface. These diagrams can be leveraged to optimize catalyst reaction conditions by precisely targeting high-activity yet stable phases. Alternatively, they can inform the necessary conditions for forming a desired surface structure, aiding in the design of synthesis conditions. By capturing the interplay between pH, electrical potential, and the actual concentration of dissolved ions, these equilibrium surface Pourbaix diagrams provide a more comprehensive and useful picture of LaMnO_3_(001) interface stability.

## Discussion

We adapted VSSR-MC to tackle the challenging case of multicomponent solid–liquid interfaces under electrical potentials and showed a pretrained machine learning force field can describe trends in surface Pourbaix grand potentials. By sampling surface reconstructions with a pretrained NFF and later fine-tuning with the generated structures, we can accurately predict surface Pourbaix diagrams and elucidate additional phases beyond those selected by human experts. The improvement of our modified VSSR-MC over alternative methods for sampling aqueous electrochemical interfaces is summarized in Table S3. Our new thermodynamic framework also respects equilibration across electrolyte species, the electrode surface, and the electrode bulk, providing a more faithful picture of aqueous electrochemical stability.

In this work, we employed the computational hydrogen electrode framework to study surface stability, but its simplified assumptions do not fully capture the solvent-mediated electrostatic interactions at the electrode–electrolyte interface.^30^ Specifically, the CHE model assumes that the electrochemical potential of aqueous species is governed solely by chemical activity, effectively setting the electrolyte as the reference for zero electrostatic potential. In reality, both the electrolyte and electrode are likely charged, forming an electric double layer that influences surface relaxation and stability predictions. Additionally, recent work^94^ highlights how shifts in Fermi energy inside the working electrode correspond to shifts in the Galvani potential, introducing surface charge effects that influence both surface thermodynamics and reaction kinetics. To capture these effects more rigorously, our framework could be extended to include solvation models and grand canonical DFT (GC-DFT) methods.^25^ VSSR-MC is compatible with such extensions because it is agnostic to the underlying energy model. For instance, a machine-learned force field could be trained on GC-DFT data to estimate surface energies and surface charges as a function of applied potential, enabling direct construction of charge-dependent Pourbaix grand potentials.^95^

We also acknowledge limitations stemming from the finite simulation cell size and our focus on a single layer of virtual sites defined above the pristine surface. While we have sampled 3 × 3 and 4 × 4 supercells, our approach does not yet encompass larger supercells or multiple layers of virtual sites, which may be necessary to capture more complex reconstructions involving deeper bulk rearrangements. Nevertheless, given that NFFs have been shown to generalize well to larger supercells,^96–98^ future expansion of the configurational space should further improve our understanding of emergent phenomena under operational conditions.

Additionally, our current framework does not explicitly account for free energy contributions from vibrational or configurational entropy of the surface slab, which could play a significant role in determining phase stability at finite temperatures.^30,99,100^ Incorporating harmonic approximations for vibrational corrections and exploring configurational entropy effects in future extensions of VSSR-MC would improve the accuracy of surface free energy predictions and better capture entropic stabilization effects.

Finally, in our surface-environment equilibria analysis, we assumed equilibrium of the aqueous dissolved species with the electrode bulk without passivation effects that may occur at the interface. We also assumed all dissolved species are of the same concentration. Additional computational and experimental studies, especially within the ORR region (roughly the bulk stability region of LaMnO_3_),^49,87^ can help us determine the physical validity of our structures and their impact in modulating ORR reactions.

## Methods

### VSSR-MC

VSSR-MC^83^ was performed in the semigrand canonical ensemble with algorithmically generated virtual surface sites. The pH and *U*_SHE_ were supplied to the simulation and the total number of adsorbates may vary across an MC run. For each semigrand VSSR-MC iteration, one adsorption site was randomly chosen to change state.

For both Pt(111) and LaMnO_3_(001), the sampling temperature was fixed at 3,000 K. For Pt(111), VSSR-MC was run at various *U*_SHE_ ∈ [0.8, 1.2] V in intervals of 0.1 V and pH ∈ [0, 12] in intervals of 4. For LaMnO_3_(001), VSSR-MC was run at various *U*_SHE_ ∈ [−1.0, 2.0] V in intervals of 0.5 V and pH ∈ [10, 14] in intervals of 2. A total of 2000 MC iterations was performed at each *U*_SHE_ and pH value. See Figures S3 and S6, respectively, for Pt(111) and LaMnO_3_(001) sampling profiles.

Following the discrete sampling step, continuous relaxation was performed using the FIRE algorithm in the ASE package.^101^ The convergence criterion was met when either a maximum of 20 relaxation steps was reached or the maximum force on all atoms fell below 0.05 eV/Å.

For each iteration in an MCMC run, the acceptance probability *P* is given by the minimum of unity and the ratio of the Boltzmann weights between the proposed and current state in Eqn. [Disp-formula eq2]

2 $$P=\min\left\{1,\exp\left(-\frac{\Delta \Omega_{surf}\left(U_{SHE},pH\right)}{k_{B}T_{sample}}\right)\right\}$$

where ΔΩ_surf_(*U*_SHE_, pH) is the change in surface Pourbaix grand potential after both discrete and continuous sampling (see “Surface Pourbaix Grand Potential” in the Methods section for details), *k*_B_ is the Boltzmann constant, and *T*_sample_ is the sampling temperature.

### Surface Slab Modeling

#### Pt­(111)

A Pt cubic unit cell from the Materials Project (mp-126) was cut in the (111) plane to create 2 × 2, 3 × 3, and $\sqrt{3}\times 3$ supercells each with four Pt layers. A vacuum spacing of 15 Å in total was set at the ends of each slab. The bottom two layers were fixed while all other atoms were allowed to relax. A total of 24 and 54 virtual adsorption sites were respectively defined for the 2 × 2 and 3 × 3 supercells at top, bridge, and hollow sites. For the $\sqrt{3}\times 3$ supercell, 6 virtual adsorption sites were defined at the top site. All sites sat at the default distance of 2.0 Å from the pristine surface using the pymatgen AdsorbateSiteFinder class. Symmetry reduction of sites was disabled. Additionally, the atomic positions of the pristine surface were included in the virtual sites.

The respective Pt virtual sites can be found in Figure S2­(a,b). For the 2 × 2 and 3 × 3 structures, Pt, O, and OH were possible adsorbates, while for $\sqrt{3}\times 3$ structures, Pt, O, OH, and H_2_O were possible adsorbates. The 8 handpicked surfaces were extracted from Hansen et al.^33^ and Vinogradova et al.^42^ Other than the pristine surface, the specific composition of each handpicked slab is documented in Figure S2­(c). These handpicked surfaces were optimized using DFT (see “Density-Functional Theory Calculations” in the Methods section).

#### LaMnO_3_(001)

A LaMnO_3_ cubic unit cell from the Materials Project (mp-19025) was cut in the (001) plane to create 2 × 2, 3 × 3, and 4 × 4 supercells with three bilayers of MnO_2_ and LaO, 6 layers each. A total vacuum spacing of 15 Å was set at the ends of each slab. The bottom three layers were fixed while all other atoms were allowed to relax. A total of 24, 54, and 96 virtual adsorption sites were respectively defined for the 2 × 2, 3 × 3, and 4 × 4 supercells at the top site. All sites sat at the default distance of 2.0 Å from the MnO_2_ pristine surface using the pymatgen AdsorbateSiteFinder class. Symmetry reduction of sites was disabled. Additionally, the atomic positions of the pristine surface were included in the virtual sites.

The 2 × 2 virtual sites can be found in Figure S5­(a,b), which extrapolate to those of the 3 × 3 and 4 × 4 supercells. For this surface, La, Mn, O, and OH were possible adsorbates. The 47 handpicked surfaces were extracted from Rong and Kolpak^49^ and modified to be asymmetric as in our choice of slab. The specific composition of each handpicked slab is documented in Figure S5­(c) and on Zenodo.^102^ These handpicked surfaces were also optimized using DFT (see “Density-Functional Theory Calculations” in the Methods section).

### Species Pourbaix Diagram Construction

The species Pourbaix diagrams for Pt and LaMnO_3_ were calculated using a combination of data retrieval and processing steps.^48,50,85^ For each material, the combined metal (non-O, non-H) species Pourbaix diagrams were constructed using a combination of DFT and experimental data obtained from the Materials Project database with the MPRester API and processed using both MaterialsProject2020Compatibility and MaterialsProjectAqueousCompatibility compatibility schemes in pymatgen to ensure data consistency. The DFT free energies of formation for stable solid species were combined with experimental ion reference data for charged dissolved species to create species Pourbaix diagrams for pH ∈ [0, 14] and *U*_SHE_ ∈ [−1.0 V, 2.0 V]. Figure S13 shows species Pourbaix diagrams of Pt and LaMnO_3_. These species Pourbaix diagrams contain multiple domains with a unique set of stable species. During sampling, the LaMnO_3_ bulk region was replaced with individual La and Mn species to allow single-atom dissolution or redeposition MC steps (Figure S13­(b)). For evaluation of thermodynamic equilibria in pH-*U*_SHE_- $c_{H_{x}{AO}_{y}^{z-}}$ , all phases were considered (Figure S14). See “Thermodynamic Equilibria” in the Methods section for details.

### Surface Pourbaix Grand Potential

We reformulated the grand potential used in VSSR-MC by adding pH and *U* dependencies: Ω_surf_(**X**, {μ}, *T*, *U*_SHE_, pH). A grand potential formulation for bulk materials under aqueous electrochemical conditions was previously proposed by refs 39,40, and 50 , but here our focus is on surfaces formed from the dominant species at each pH and *U*_SHE_. The grand potential of any slab was calculated in two steps, inspired by the adsorption-dissolution framework described in refs 49,56 and generalizes the single-atom dissolution process in Figure 1(a) to compute an absolute energy.

1. All atoms were dissociated from the surface slab to their standard states, i.e., bulk for metal atoms, *H*_2_(*g*) for hydrogen, and *O*_2_(*g*) for oxygen. This energy change is computed with Eqn. [Disp-formula eq3]
3 $$\Omega_{1}=\underset{A\in A}{\sum }N_{A}\mu_{A}^{⊖}-G_{slab}$$
  where *G*_slab_ is the Gibbs free energy of the slab, A is the set of elements present in the slab, *N*_A_ is the number of atoms of element A in the slab, μ_A_^⊖^ is the standard state chemical potential of element A. *G*_slab_ was estimated with the slab energy calculated using the prescribed energy model, e.g., DFT or NFF. Raw DFT energies were corrected with MaterialsProject2020Compatibility. Additionally, a zero-point energy-TS (ZPE-TS) correction of 0.23 eV was added to each OH adsorbate^49^ on the slab. μ_A_^⊖^ values were obtained with MPRester and then corrected with pymatgen.
2. Each standard state species A was reacted to form the most stable species under aqueous electrochemical conditions with the general formula H_*x*_AO_*y*_^*z* ^–^^, where *x*, *y*, and *z* are integer coefficients. The chemical equation for each individual A is in Eqn. [Disp-formula eq4]
4 $$A+N_{A,H_{2}O}H_{2}O\to H_{x}AO_{y}^{z^{-}}+N_{A,H^{+}}H^{+}+N_{A,e}e^{-}$$
  where $N_{A,H_{2}O}$ , $N_{A,H^{+}}$ , and *N*_A,e_ are the stoichiometric coefficients of H_2_O, H^+^, and e^–^ in the reaction, respectively. The dominant aqueous electrochemical species A depends on the pH, *U*_SHE_, *T*, and $\mu_{H_{x}{AO}_{y}^{z-}}$ . For this work, we evaluated energies at *T* = 298 K and $\mu_{H_{x}{AO}_{y}^{z-}}=\mu_{H_{x}{AO}_{y}^{z-}}^{⊖}+k_{B}T\;\ln a_{H_{x}{AO}_{y}^{z-}}$ , where $\mu_{H_{x}{AO}_{y}^{z-}}^{⊖}$ is the standard state energy of the species retrieved from the Materials Project database and $a_{H_{x}{AO}_{y}^{z-}}$ is the species activity, which we assume to be 1 for solid species and 10^–6^ for dissolved species unless specified. The most stable species was queried at each set of pH and *U*_SHE_, which effectively is the dominant phase present in the species Pourbaix diagrams (see previous section). The total energy change for step 2 is computed with Eqn. [Disp-formula eq5]
5 $$\Omega_{2}=\underset{A\in A}{\sum }N_{A}\left[\mu_{H_{x}{AO}_{y}^{z-}}+N_{A,H^{+}}\mu_{H^{+}}+N_{A,e}\mu_{e}-\mu_{A}^{⊖}-N_{A,H_{2}O}\mu_{H_{2}O}^{⊖}\right]=\underset{A\in A}{\sum }N_{A}\left[\Delta \Omega_{A,SHE}^{⊖}-2.3N_{A,H^{+}}k_{B}TpH-N_{A,e}\left(eU_{SHE}\right)+k_{B}T\;\ln a_{H_{x}{AO}_{y}^{z-}}\right]$$
  where ΔΩ_A,SHE_^⊖^ is the standard-state free energy of reaction for species A under SHE and is in accordance with the CHE formulation. Similarly, ΔΩ_A,SHE_^⊖^ was derived from existing energies queried from the Materials Project without additional first-principles calculations. Tables S1 and S2 show ΔΩ_A,SHE_^⊖^ and $\Delta \Omega_{A,SHE}^{⊖}-2.3N_{A,H^{+}}k_{B}TpH-N_{A,e}\left(eU_{SHE}\right)+k_{B}T\;\ln a_{H_{x}{AO}_{y}^{z-}}$ for Pt(111) and LaMnO_3_(001), respectively. Comprehensive data is also included in our Zenodo data set.^102^

The surface Pourbaix grand potential is defined as the formation energy from the stable aqueous species, Ω_surf_(**X**, {μ}, *T*, *U*_SHE_, pH) = −(Ω_1_ + Ω_2_), with the negative sign.

### Surface Analysis

#### Pt­(111)

Following each VSSR-MC run, the sampled surfaces were initially downselected to 100 structures from the original 2000 generated per run. 2 × 2 and 3 × 3 structures with more than 4 and 9 O atoms, respectively, were filtered out to mitigate excessive O_2_ adsorption introduced by MaterialsProject2020Compatibility corrections, yielding 1131 and 979 structures, respectively. All 2000 $\sqrt{3}\times 3$ structures were initially included. After removing duplicate Pt(111) pristine surfaces, a total of 3964 unique surfaces, along with the 8 handpicked structures, were evaluated using fine-tuned MACE to generate the relative surface Pourbaix grand potential plot in Figure S9.

#### LaMnO_3_(001)

Following each VSSR-MC run, the sampled surfaces were also downselected to 100 structures from the original 2000 generated per run. 2 × 2 structures with 8 or fewer Mn atoms but more than 38 O atoms were filtered out to mitigate excessive O_2_ adsorption caused by MaterialsProject2020Compatibility corrections, yielding 1319 structures. An additional 7 structures were removed due to unphysical bonding configurations, such as floating O* or OH* on oxygen atoms. The remaining 1312 surfaces, along with the 47 handpicked surfaces, were then evaluated using fine-tuned CHGNet to generate the relative surface Pourbaix grand potential plots in Figures 4(a) and S11­(a). Meanwhile, 3 × 3 and 4 × 4 structures from *U*_SHE_ ∈ [−1.0 V, 0.5 V] were selected without additional filtering to gather 1200 structures for each size, which were evaluated using fine-tuned CHGNet to generate the relative surface Pourbaix grand potential plots in Figure S11­(b,c), respectively.

Subsequently, 27 low-energy 2 × 2 structures were selected based on their fine-tuned CHGNet ΔΩ_surf_(*U*_SHE_, pH) values at *U*_SHE_ = 0.6 V and pH = 12, specifically those at most 0.1 eV/surface unit cell (6.42 meV/Å^2^) higher in energy than the initially most stable surface, MnO_2_- $\frac{1}{4}$ OH*. These structures were further relaxed with DFT, resulting in 23 surfaces that were more stable at the DFT level than MnO_2_- $\frac{1}{4}$ OH* under the same conditions (Figure S10). Integrating these structures with those from literature for convex hull analysis resulted in the updated surface Pourbaix diagram shown in Figure 4(b).

### Thermodynamic Equilibria

To extend the 2D species and surface Pourbaix diagrams from pH-*U*_SHE_ space into 3D pH-*U*_SHE_- $c_{H_{x}{AO}_{y}^{z-}}$ space, we allowed the chemical potentials of dissolved species to vary with concentration. In particular, the species chemical potential, $\mu_{H_{x}{AO}_{y}^{z-}}$ , is related to its activity, $a_{H_{x}{AO}_{y}^{z-}}$ , through $\mu_{H_{x}{AO}_{y}^{z-}}=\mu_{H_{x}{AO}_{y}^{z-}}^{⊖}+k_{B}T\;\ln a_{H_{x}{AO}_{y}^{z-}}$ and under dilute concentrations ( $c_{H_{x}{AO}_{y}^{z-}}\leq 10^{-3}$ M), the activity can be approximated by the concentration $a_{H_{x}{AO}_{y}^{z-}}\approx c_{H_{x}{AO}_{y}^{z-}}$ .

Using this relationship, we performed convex hull analyses over a four-dimensional (4D) space (pH-*U*_SHE_- $c_{H_{x}{AO}_{y}^{z-}}$ and the energy) to construct 3D Pourbaix diagrams (Figures S14, S15 and S16). First, we generated the 3D *species* Pourbaix diagram with convex hull analysis to determine the dominant species A across pH-*U*_SHE_- $c_{H_{x}{AO}_{y}^{z-}}$ space. Next, we expressed the *surface* Pourbaix grand potential as a function of an additional variable, Ω_surf_({*c*}, *U*_SHE_, pH), where {*c*} represents the set of concentrations for each dominant dissolved species, $c_{H_{x}{AO}_{y}^{z-}}$ . We do this analysis for each set of dominant species, A, as in Figure S1, and separately for both Figures S15 and S16. A second round of convex hull analyses in 4D space yielded the 3D *surface* Pourbaix diagrams.

These 3D Pourbaix diagrams have independent pH, *U*_SHE_, and $c_{H_{x}{AO}_{y}^{z-}}$ axes and thus are not under full equilibration between the electrode bulk, electrode surface, and electrolyte. To achieve full equilibrium, each species neighboring the LaMnO_3_ bulk phase in the *species* Pourbaix diagram must be in equilibrium with the bulk itself, i.e., at the phase boundaries where they meet. For example, consider La^3+^ + MnO_2_(*s*) (electrolyte) in equilibrium with LaMnO_3_(*s*) (electrode) at 10^–6^ M dissolved species concentrations in Figure S13­(b). The energy changes from standard-state species were evaluated using Eqns. [Disp-formula eq4] and 5 and set equal, leading to the equilibrium condition in Eqn. [Disp-formula eq6]:

6 $$\mu_{{LaMnO}_{3}}+2\cdot 2.3k_{B}TpH=\mu_{{La}^{3+}}+\mu_{{MnO}_{2}}+\mu_{H_{2}O}+eU_{SHE}$$

Here, μ_LaMnO_3__ ≈ *E*_LaMnO_3__^DFT^, μ_MnO_2__ ≈ *E*_MnO_2__^DFT^, and $\mu_{H_{2}O}\approx -2.46\;eV/H_{2}O$ . This equation leaves three adjustable parameters (pH, *U*_SHE_, and *c*_La^3+^_) but only two degrees of freedom. By applying analogous equilibrium conditions for every species adjacent to LaMnO_3_ in the 3D *species* Pourbaix diagram (pH ∈ [0, 14], *U*_SHE_ ∈ [−1.0 V, 2.0 V], and $c_{H_{x}{AO}_{y}^{z-}}\in \left[10^{-15}\;M,10^{-3}\;M\right]$ ), we effectively trace out the LaMnO_3_ bulk stability boundaries and label the neighboring species at each point, leading to the equilibrium *species* Pourbaix diagram where pH, *U*_SHE_, and $c_{H_{x}{AO}_{y}^{z-}}$ are thermodynamically coupled. In practice, we introduced a slightly offset hyperplane representing the LaMnO_3_ phase in the 4D space, oriented opposite to the original LaMnO_3_ plane.

Finally, we traced out the same bulk stability boundaries from the 3D *surface* Pourbaix diagrams in Figures S15 and S16 to enforce full electrode-surface-electrolyte equilibrium and obtain the equilibrium *surface* Pourbaix diagrams, with pH, *U*_SHE_, and $c_{H_{x}{AO}_{y}^{z-}}$ also thermodynamically coupled. This approach parallels the well-established framework for thermodynamic equilibria in contact with gas.^83,99,103–106^ However, unlike gas–solid interfaces where species identities are fixed, aqueous species depend on pH, *U*_SHE_, and $c_{H_{x}{AO}_{y}^{z-}}$ .

### Active Learning

For Pt(111), the medium MACE-MP-0 pretrained model^90^ was fine-tuned for 60 epochs. The loss function was a weighted sum of the mean-squared errors of forces and energy with a 100:1 ratio for weights of forces and energies, respectively. The node embedding, pooling, and readout layers were fully unfrozen while for the interaction layers only the linear layers were allowed to changed.

For LaMnO_3_(001), pretrained CHGNet^67^ v0.3.0 was fine-tuned for 60 epochs. The loss function was a weighted sum of the mean-squared errors of forces and energy with a 100:5 ratio of weights. For pretrained CHGNet, unfreezing the readout layer was sufficient for good fine-tuning performance on LaMnO_3_(001). For both systems, raw DFT energies were corrected with the MaterialsProject2020Compatibility scheme and the Adam optimizer^107^ was used with a learning rate of 0.001.

Structures were selected for active learning using latent space clustering, as in Du et al.^83^ Briefly, the VSSR-MC generated structures for each surface were clustered according to the first three principal components (PCs; >90% explained variance) of their NFF embeddings and the most uncertain structure for each cluster was selected. To save on compute time, a first-pass clustering was run for every 1,000 samples to yield around 100–200 structures at each set of pH and *U*_SHE_. The results of one full clustering run for each of Pt(111) and LaMnO_3_(001) is shown in Figure S18. For Pt(111), 115 sampled structures were eventually used for fine-tuning. For LaMnO_3_(001), the number was 118.

The metric for uncertainty quantification was changed to Gaussian-mixture model (GMM) uncertainty, which is more suitable for a single NFF model used here.^108^ The GMM model was calibrated against the force MAE of a 5000-structure subset randomly selected from MPtrj,^89^ which was used for training the foundational models. We show the uncertainty vs force MAE plot in Figure S19 for the MPtrj calibration data set and all sizes of Pt(111) and LaMnO_3_(001) fine-tuning structures. The performance is commensurate with previous work.^108,109^

Additionally, Pt(111) and LaMnO_3_(001) structures in the pretrained CHGNet surface Pourbaix diagrams were rattled by randomly displacing atoms in all three spatial directions, with a maximum displacement of ± 0.1 Å, to better sample the near-equilibrium potential energy surface. A single active learning cycle was sufficient to refine the respective NFF models, reducing their MAEs to within 10 meV/atom relative to DFT references across all surfaces, as validated against handpicked literature structures (see Figures S4 and S7). For Pt(111), 40 additional structures were obtained in this way, while for LaMnO_3_(001), 18 more structures were acquired.

### Density-Functional Theory Calculations

Vienna *ab initio* Simulation Package (VASP) v.6.2.1 ^110,111^ was employed for both single-point DFT calculations and DFT relaxations using the projector augmented-wave (PAW) method to describe core electrons.^112,113^ The following PAW data sets version 54 pseudopotentials were used: H, O, Pt, La, and Mn_pv. The Perdew–Burke–Ernzerhof (PBE) functional^114^ version of the generalized-gradient approximation (GGA) was used for all calculations. Grimme’s D3 method with Becke-Johnson damping was used to account for dispersion interactions.^115,116^ All calculations were spin-polarized. Additionally, DFT+U calculations were used for LaMnO_3_(001) applying Dudarev’s approach^117^ with the single on-site parameter for Mn set to 3.9 on the d-orbital electrons, consistent with the Materials Project settings.^118^ Dipole corrections to the total energy were enabled along the *z*-axis for surfaces. The kinetic energy cutoff for plane waves was set to 520 eV. Integrations over the Brillouin zone were performed using a 6 × 6 × 1 Gamma centered mesh for 2 × 2 Pt(111), 4 × 4 × 1 Gamma centered mesh for 3 × 3 Pt(111), 6 × 4 × 1 Gamma centered mesh for $\sqrt{3}\times 3$ Pt(111), and a 3 × 3 × 1 Gamma centered mesh for LaMnO_3_(001). In the self-consistent field cycle, a total energy limit of 10^–6^ eV was adopted as the stopping criterion.

For surfaces that were optimized, optimization of atomic positions was performed until the Hellmann–Feynman forces on atoms were smaller than 10 meV/Å or until 100 relaxation steps were reached using the conjugate gradient algorithm for optimization and for electronic minimization within each self-consistent field calculation. After 100 relaxation steps, any unconverged surfaces were further relaxed with the RMM-DIIS algorithm until the change in total energy between two relaxation steps was smaller than 10 meV.

### Workflow Management and Compute Time

An internal library, HTVS (for high-throughput virtual simulations) managed the DFT calculations. VSSR-MC and active learning were run in separate procedures. DFT single-point calculations took about 15 min to 1 h each on an NVIDIA Volta V100 32 GB GPU (MIT SuperCloud) while relaxations took about 3–6 h each on an NVIDIA Tesla A100 Ampere 40 GB GPU (NERSC Perlmutter). VSSR-MC runs were 2000 iterations each. For Pt(111), 2 × 2 slab runs took 30 min each, 3 × 3 slab runs took 50 min each, while $\sqrt{3}\times 3$ slab runs took 40 min each on an NVIDIA GeForce RTX 2080 Ti 11 GB GPU. Meanwhile, for LaMnO_3_(001), 2 × 2 slab runs took 70 min each, 3 × 3 slab runs took 140 min each, while 4 × 4 slab runs took 210 min each also on an NVIDIA GeForce RTX 2080 Ti 11 GB GPU. Active learning was relatively fast, with latent-space clustering and fine-tuning taking 15 min or less on the same NVIDIA GeForce RTX 2080 Ti 11 GB GPU.

## Supplementary Material





## Data Availability

The fine-tuned models, DFT data, selected results from VSSR-MC runs, and Jupyter notebooks used for data analysis and plotting are available on Zenodo: 10.5281/zenodo.15066440. The VSSR-MC algorithm reported in this work is available on GitHub: https://github.com/learningmatter-mit/surface-sampling. Our version of pymatgen for constructing surface Pourbaix and 3D Pourbaix diagrams, along with additional plotting methods, is also available on GitHub: https://github.com/xiaochendu/pymatgen.

## Notes

(1) A list of abbreviations and surface nomenclature conventions; (2) additional analysis of LaMnO_3_(001) surface Pourbaix diagrams; (3) supplementary figures, including fine-tuning results and sampling profiles for Pt(111) and LaMnO_3_(001), schematics for surface Pourbaix diagram construction, handpicked and sampled surface structures, surface energies, surface Pourbaix diagrams across supercell sizes and species concentrations, species Pourbaix diagrams, 3D visualizations of stability regions, and clustering and uncertainty quantification results; and (4) supplementary tables comparing alternative sampling methods and listing free energies of reaction for relevant species (PDF)
Transparent Peer Review report available (PDF)
