# Supplementary material for: Accelerating and Enhancing Thermodynamic Simulations of Electrochemical Interfaces
Source: ACS Cent Sci. 2025 Jul 21;11(9):1558–72. doi: 10.1021/acscentsci.5c00547 (PMC12464767; doi:10.1021/acscentsci.5c00547)
Supplement: Supplementary file 1 [file oc5c00547_si_001.pdf]

Supporting Information: Accelerating and enhancing thermodynamic simulations of electrochemical interfaces

Xiaochen Du,[†] Mengren Liu,[‡] Jiayu Peng,[‡] Hoje Chun,[‡] Alexander Hoffman,[‡] Bilge Yildiz,[‡,¶] Lin Li,[§] Martin Z. Bazant,[†] and Rafael Gómez-Bombarelli[*,‡]

†*Department of Chemical Engineering, Massachusetts Institute of Technology, Cambridge, MA 02139, USA*

‡*Department of Materials Science and Engineering, Massachusetts Institute of Technology, Cambridge, MA 02319, USA*

¶*Department of Nuclear Science and Engineering, Massachusetts Institute of Technology, Cambridge, MA 02319, USA*

§*Massachusetts Institute of Technology Lincoln Laboratory, Lexington, MA 02421, USA*

E-mail: rafagb@mit.edu

Abbreviations used

- CHE: Computational hydrogen electrode
- DFT: Density-functional theory
- GC-DFT: Grand canonical density-functional theory
- GCMC: Grand canonical Monte Carlo
- GGA: Generalized-gradient approximation
- GMM: Gaussian mixture model
- MAE: Mean absolute error
- MC: Monte Carlo
- MCMC: Markov-chain Monte Carlo
- ML: Machine learning
- NFF: Neural network force field
- OER: Oxygen evolution reaction
- ORR: Oxygen reduction reaction
- PAW: Projector augmented-wave
- PBE: Perdew-Burke-Ernzerhof (functional)
- PC: Principal component
- SHE: Standard hydrogen electrode
- VASP: Vienna *ab initio* Simulation Package
- VSSR-MC: Virtual Surface Site Relaxation-Monte Carlo
- ZPE-TS: Zero-point energy minus temperature-entropy correction

Surface nomenclature

Pt(111) We denote the adlayer of a given adsorbate with the fractional coverage (θ, the number of adsorbates per exposed metal atom) preceding the adsorbate of interest (e.g., $\frac{1}{4}$O*).

$LaMnO_3$(001) We denote the surface structure by the stoichiometry of the surface termination, the fractional coverage of the prevailing adsorbate across sites, and the adsorbate species. For example, an MnO_2 termination with $\frac{1}{4}$ of all metal sites covered with OH* is denoted MnO_2-$\frac{1}{4}$OH*, with a hyphen used for clarity. Meanwhile, vacancies and substitutions in the pristine surface layer are denoted by 'vac' and 'sub' subscripts respectively.

Additional $LaMnO_3$(001) surface Pourbaix analysis

Surface Pourbaix diagram trends

At oxidizing conditions, surfaces are predominantly terminated by MnO_2- or Mn_{vac}-; in more reducing conditions at lower U_{SHE}, LaO-type terminations become more preferred. Within each termination type, O* predominantly adsorbs at high U_{SHE} and pH, while OH* adsorption (protonation of O*) becomes prevalent under more reducing conditions.

Fine-tuning results analysis

The key differences between the pre-trained CHGNet (Fig. 3(b)) and the DFT (Fig. 3(a)) surface Pourbaix diagrams are the replacement of $\frac{3}{4}Mn_{vac}$ in the top right (high U_{SHE} and high pH) of the DFT diagram with LaO-O* in the pre-trained CHGNet diagram, along with the emergence of an additional $\frac{1}{4}Mn_{vac}$ region in Fig. 3(b).

Since pre-trained CHGNet predicted the surface Pourbaix diagram fairly well (Fig. 3(b)), we only fine-tuned CHGNet for $LaMnO_3$(001). After fine-tuning, we re-evaluated energies

for the handpicked structures and re-plotted the surface Pourbaix diagram in Fig. 3(c). This Pourbaix diagram has all but one phase agreeing with (the exception being a thin strip of $\frac{1}{4}\mathrm{Mn}_{\mathrm{vac}}$ present in Fig. 3(c)) and phase boundaries more aligned with the DFT reference, especially closer to the sampled bulk stability region with which we are concerned.

We plot $\Delta\Omega_{\mathrm{surf}}(U_{\mathrm{SHE}}, \mathrm{pH})$ with respect to the pristine MnO_2-terminated surface against U_{SHE} for the dominant surface phases by taking a slice along pH = 12 (Fig. S8(a-c)). As reflected in the surface Pourbaix diagrams, we find that most energies for the pre-trained CHGNet (Fig. S8(b)) are well-aligned with the DFT reference for $LaMnO_3$(001) (Fig. S8(a)) with a 0.095 eV/atom energy MAE (Fig. S7(a)). This behavior contrasts with that of Pt(111), where pre-trained CHGNet was only able to predict qualitative trends with a much worse energy MAE of 0.531 eV/atom (Fig. S4(a)). Only the energies of LaO-$\frac{1}{2}$OH* with respect to the pristine MnO_2 and $\frac{1}{4}\mathrm{Mn}_{\mathrm{vac}}$ with respect to MnO_2-$\frac{1}{4}$OH* are underestimated.

After fine-tuning, we observe in Fig. S8(c) a large improvement in the relative surface Pourbaix grand potential of LaO-$\frac{1}{2}$OH* (from 24.4 meV/Å^2 to 47.2 meV/Å^2 at $U_{\mathrm{SHE}} = 0$) and a slight improvement in the relative surface Pourbaix grand potential of $\frac{1}{4}\mathrm{Mn}_{\mathrm{vac}}$ (from 20.2 meV/Å^2 to 21.3 meV/Å^2 at $U_{\mathrm{SHE}} = 0$) to be closer to the DFT values in Fig. S8(a). We highlight that small changes of 1's or 10's of meV/Å^2 is sufficient to change the relative ordering of surface stability, as in $\frac{1}{4}\mathrm{Mn}_{\mathrm{vac}}$, and thus alter the resultant surface Pourbaix diagram; nevertheless, our fine-tuned NFF can very closely match the relatively-complicated DFT reference surface Pourbaix diagram for $LaMnO_3$(001).

Supplementary figures

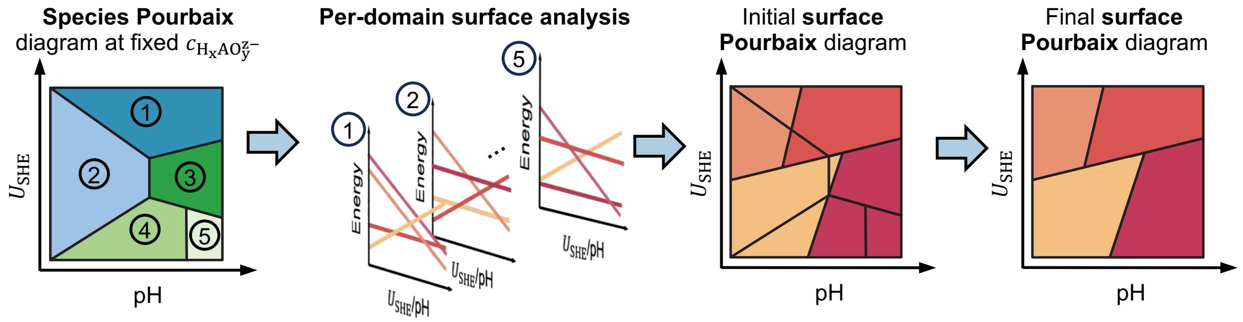

Figure S1: **Schematic for conventional surface Pourbaix diagram at fixed $c_{\mathrm{H_xAO_y^{z-}}}$.** Separate $\Omega_{\mathrm{surf}}(U_{\mathrm{SHE}}, \mathrm{pH})$ is considered for each surface in each species domain. Individual convex hull analyses yield stable surface domains for each species domain. Merging identical surface domains results in the final surface Pourbaix diagram.

.

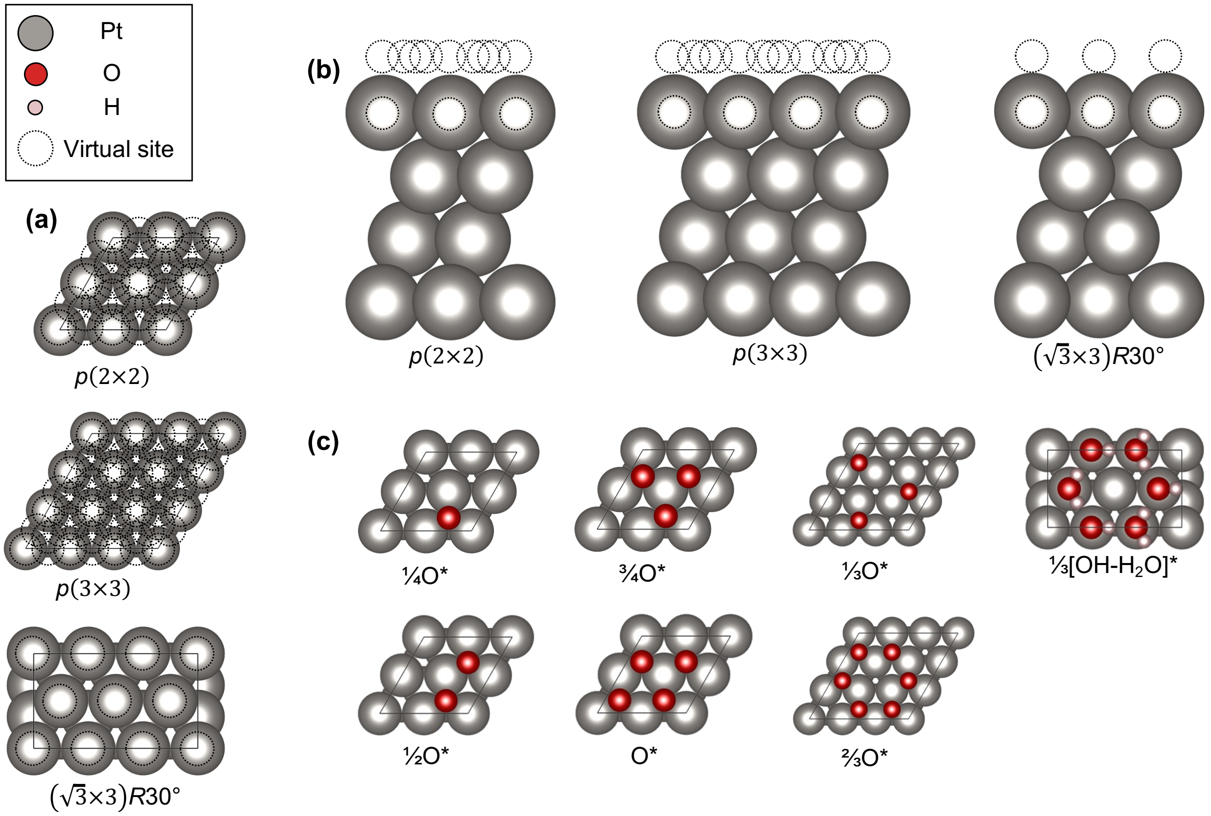

Figure S2: **Set of literature surfaces used in Pt(111) surface analysis.** (a) Top-down view of $(\sqrt{3}\times3)$R30°, $p(2\times2)$, and $p(3\times3)$ pristine surfaces and virtual sites used for VSSR-MC sampling. (b) Side view of pristine surfaces and virtual sites. The pristine surface layer is included in the virtual sites. (c) Handpicked reconstructions obtained from Hansen *et al.*[S1] and Vinogradova *et al.*[S2]

.

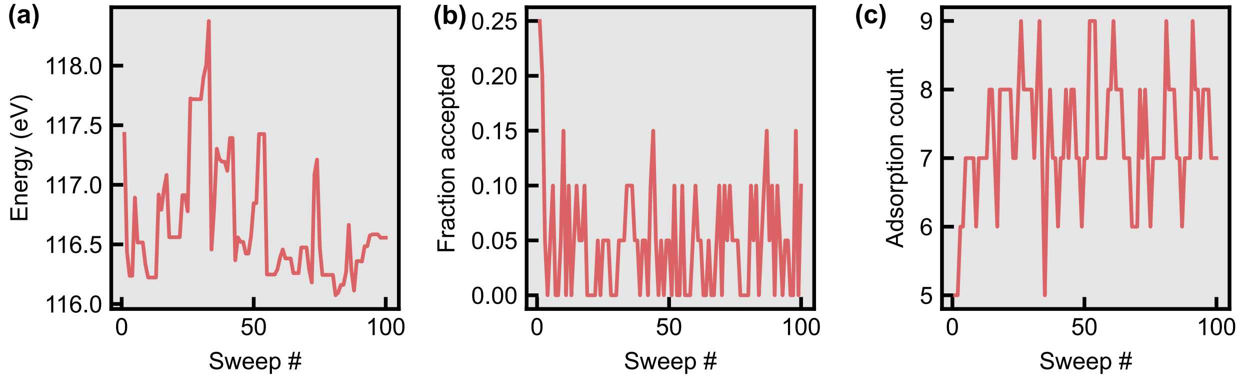

Figure S3: **VSSR-MC sampling profile for** $p(3\times3)$ **Pt(111) at pH = 8,** $U_{\mathbf{SHE}}$ **= 1.0 V.** (a) Surface Pourbaix grand potential ($\Omega_{\mathrm{surf}}(U_{\mathrm{SHE}},\mathrm{pH})$) energy evaluated using pretrained CHGNet, (b) fraction of MC iterations accepted, and (c) number of adsorbed atoms. Each sweep consists of 20 MC sampling iterations.

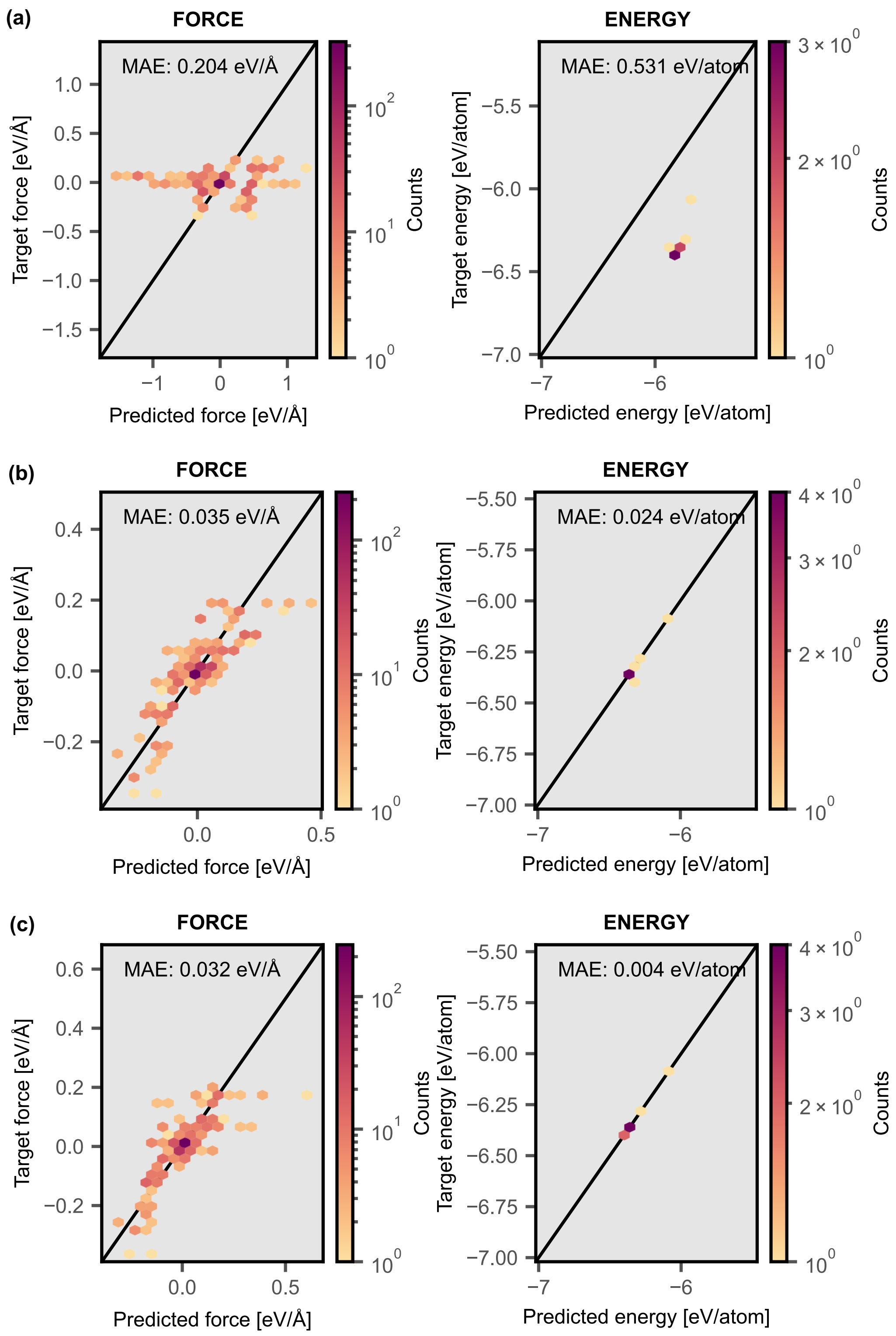

Figure S4: **NFF performance comparison for Pt(111) on the 8 handpicked surfaces.** (a) pre-trained CHGNet, (b) fine-tuned CHGNet, and (c) fine-tuned MACE.

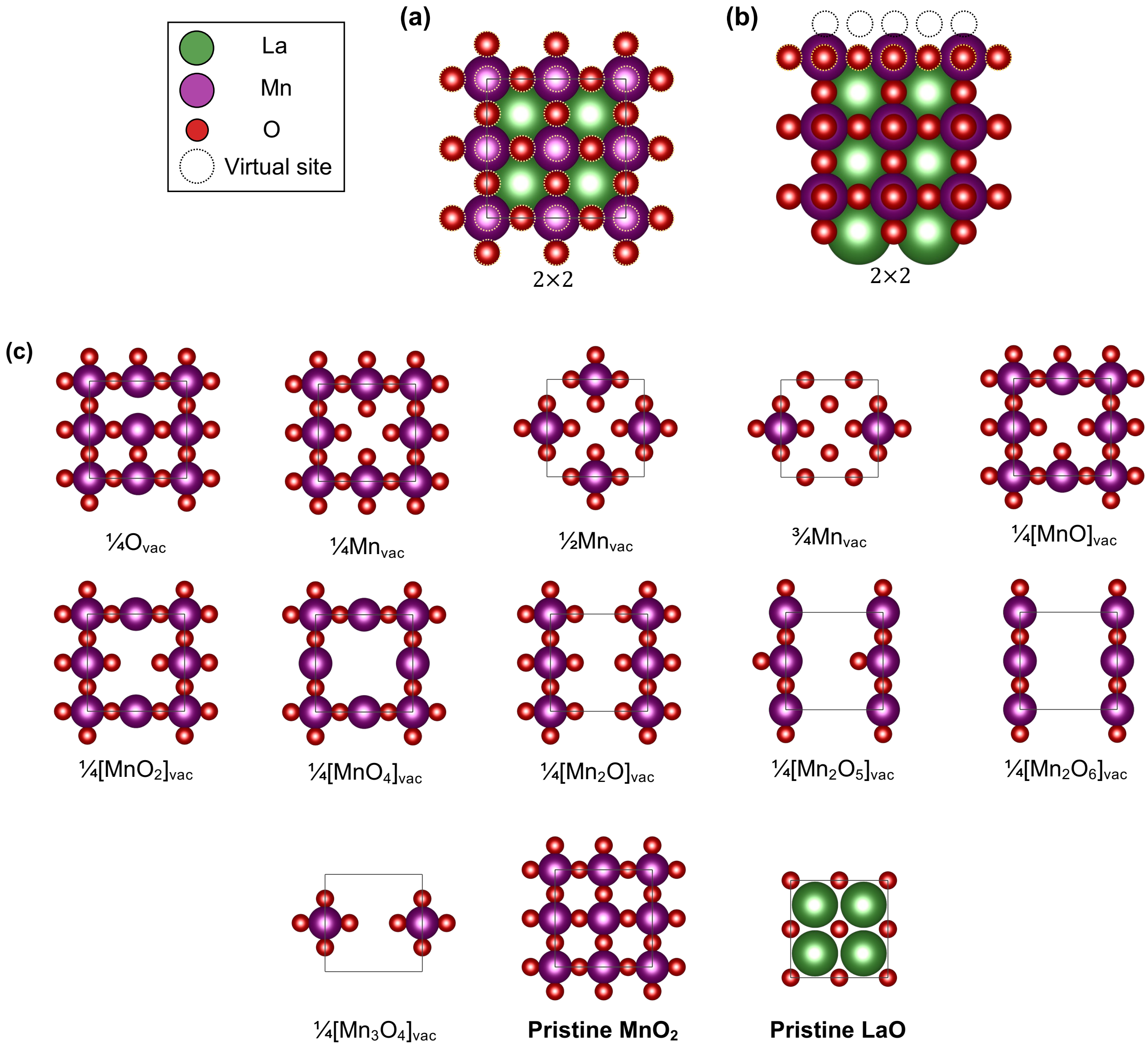

Figure S5: **Set of literature surfaces used in $LaMnO_3$(001) surface analysis.** (a) Top-down view of 2×2 pristine surface and virtual sites used for VSSR-MC sampling. (b) Side view of pristine surface and virtual sites. The pristine surface layer is included in the virtual sites. (c) Handpicked reconstructions obtained from Rong and Kolpak.[S3] Additional surfaces with varying coverages of O* and OH* were also included for a total of 47 structures.[S4]

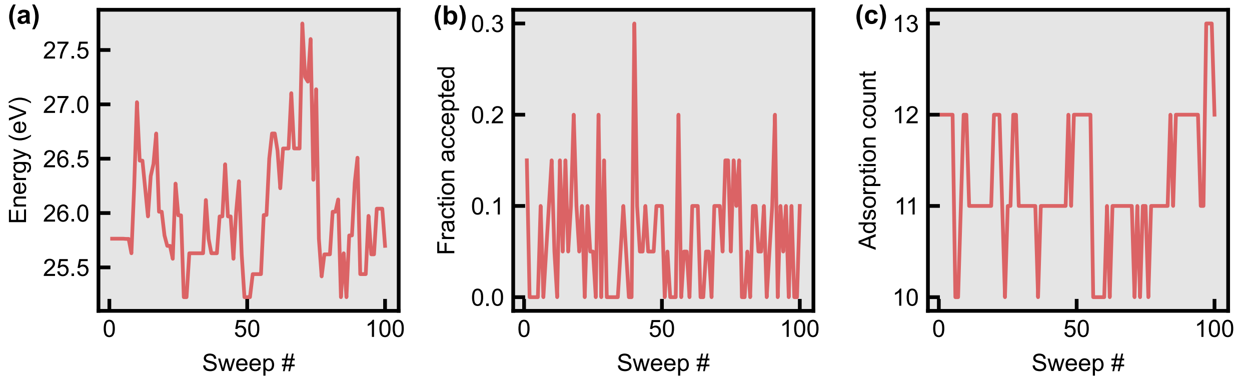

Figure S6: **VSSR-MC sampling profile for 2×2 $\mathbf{LaMnO_3}$(001) at pH = 10, $U_{\mathbf{SHE}}$ = 1.0 V.** (a) Surface Pourbaix grand potential ($\Omega_{\mathrm{surf}}(U_{\mathrm{SHE}}, \mathrm{pH})$) energy evaluated using pre-trained CHGNet, (b) fraction of MC iterations accepted, and (c) number of adsorbed atoms. Each sweep consists of 20 MC sampling iterations.

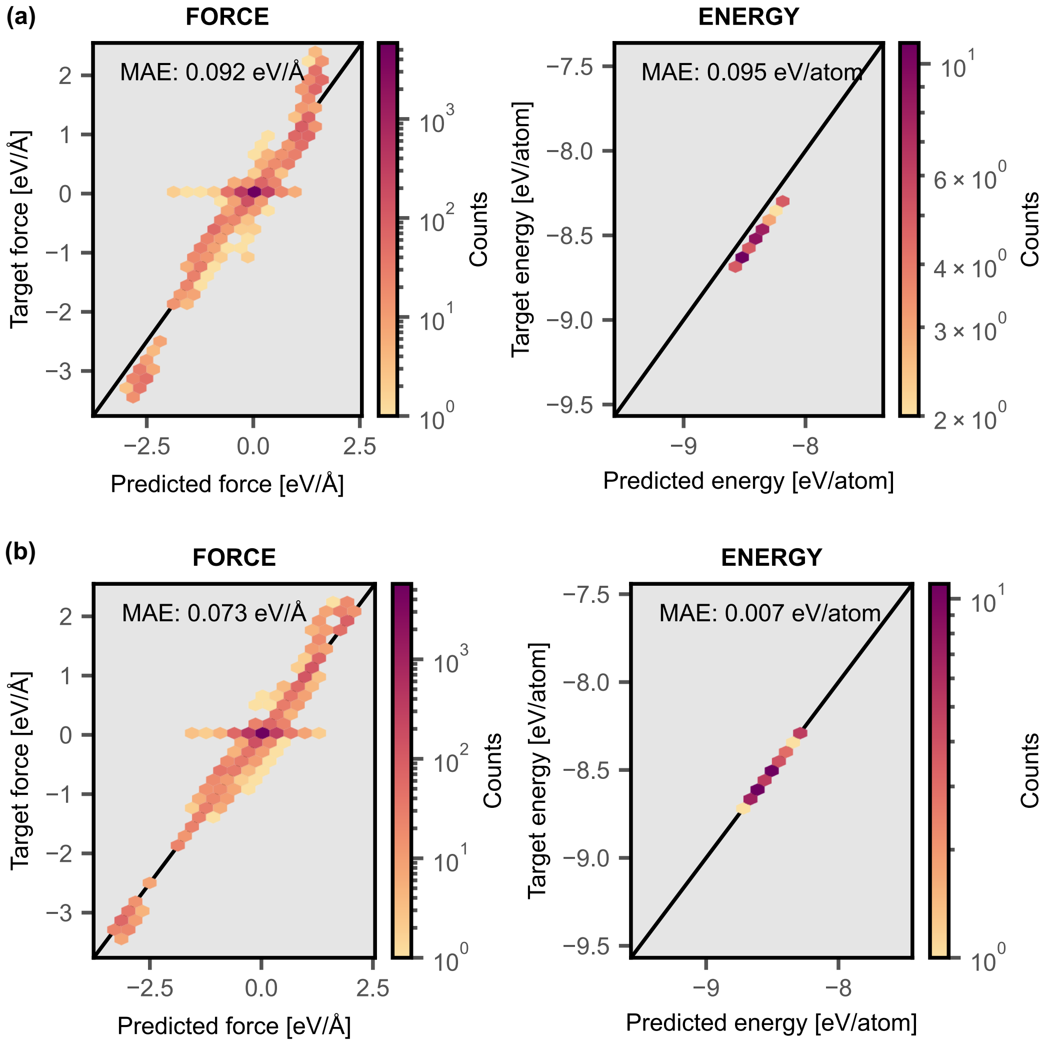

Figure S7: **NFF performance comparison for $LaMnO_3$(001) on the 47 handpicked surfaces.** (a) pre-trained CHGNet and (b) fine-tuned CHGNet.

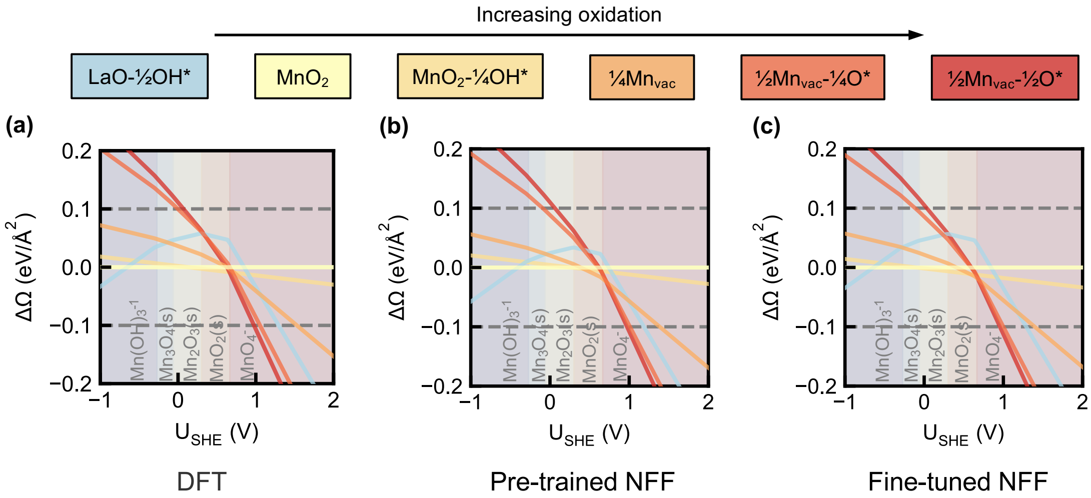

Figure S8: **LaMnO$_3$(001) surface energy analysis with various energy models.** $\Delta\Omega_{\mathrm{surf}}(U_{\mathrm{SHE}}, \mathrm{pH})$ comparison of handpicked LaMnO$_3$(001) surfaces with respect to the pristine surface in eV/Å^2 across U_{SHE} at fixed pH = 12 with (d) DFT, (e) pre-trained NFF, and (f) fine-tuned NFF energies. The color sequence approximately follows increasing oxidation level. The stable species domains are differentiated through the background color, with La^{+3} as the dominant La species and the dominant Mn species labeled in gray. Dashed lines are a guide for the eye.

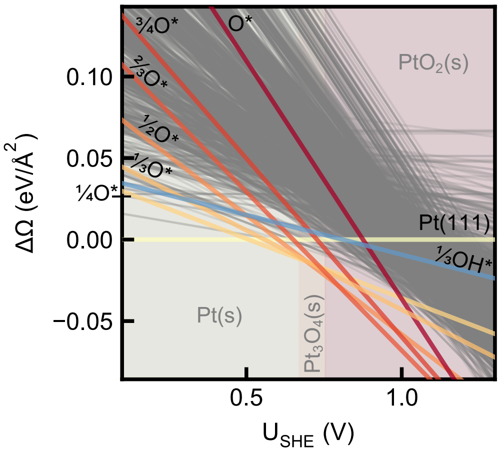

Figure S9: **Pt(111) energy analysis including additional sampled structures in gray.** $\Delta\Omega_{\mathrm{surf}}(U_{\mathrm{SHE}}, \mathrm{pH})$ with respect to the pristine surface in eV/Å^2 of handpicked and VSSR-MC sampled structures evaluated with fine-tuned MACE energies across U_{SHE} at fixed pH = 0.

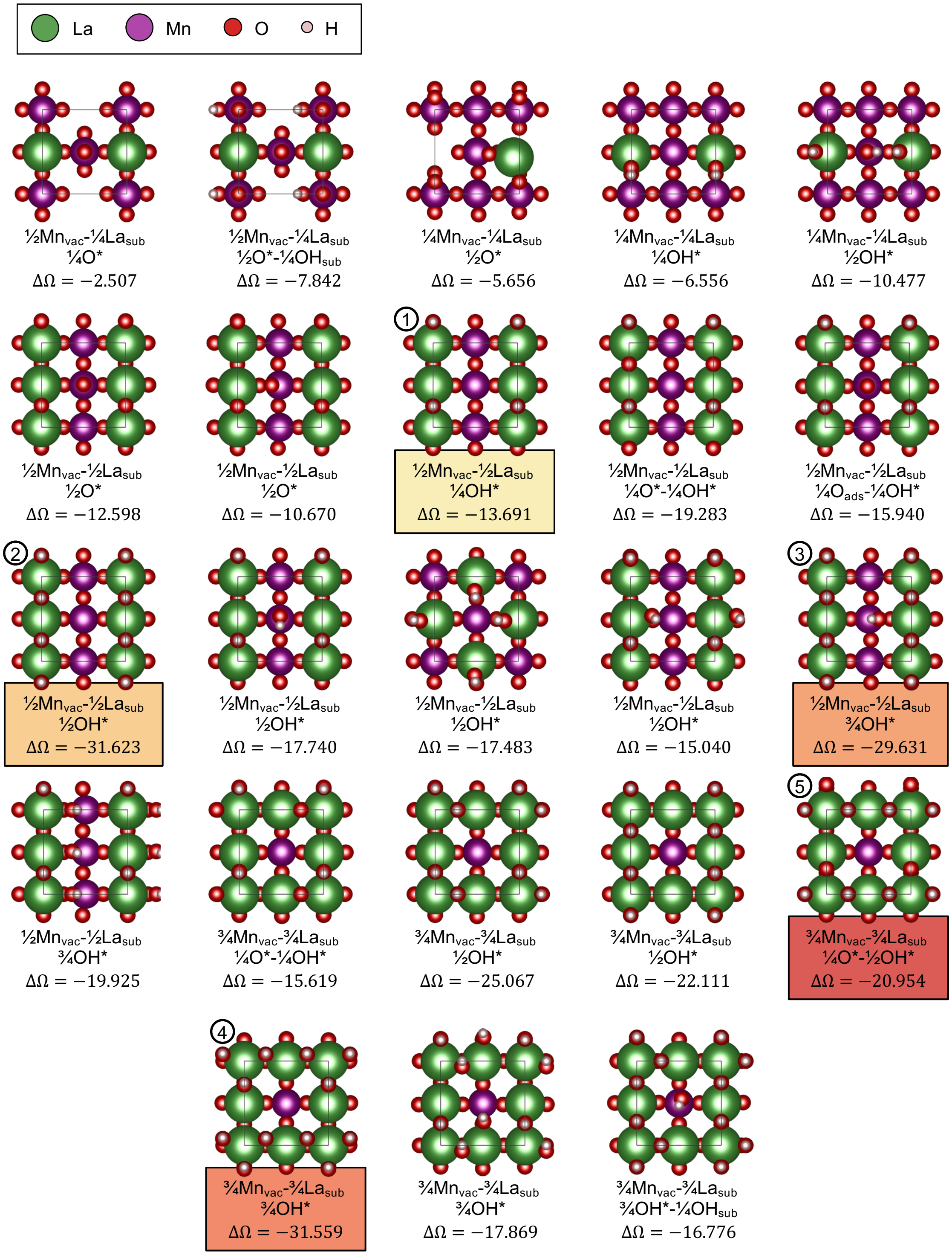

Figure S10: **Sampled mixed termination $LaMnO_3$(001) surfaces more stable than MnO_2-$\frac{1}{4}$OH* at U_{SHE} = 0.6 V, pH = 12.** $\Delta\Omega_{surf}(U_{SHE}, \mathrm{pH})$ energies are evaluated at the DFT level with respect to MnO_2-$\frac{1}{4}$OH* in eV/Å^2. Highlighted surfaces correspond to stable domains in Fig. 4(b-c).

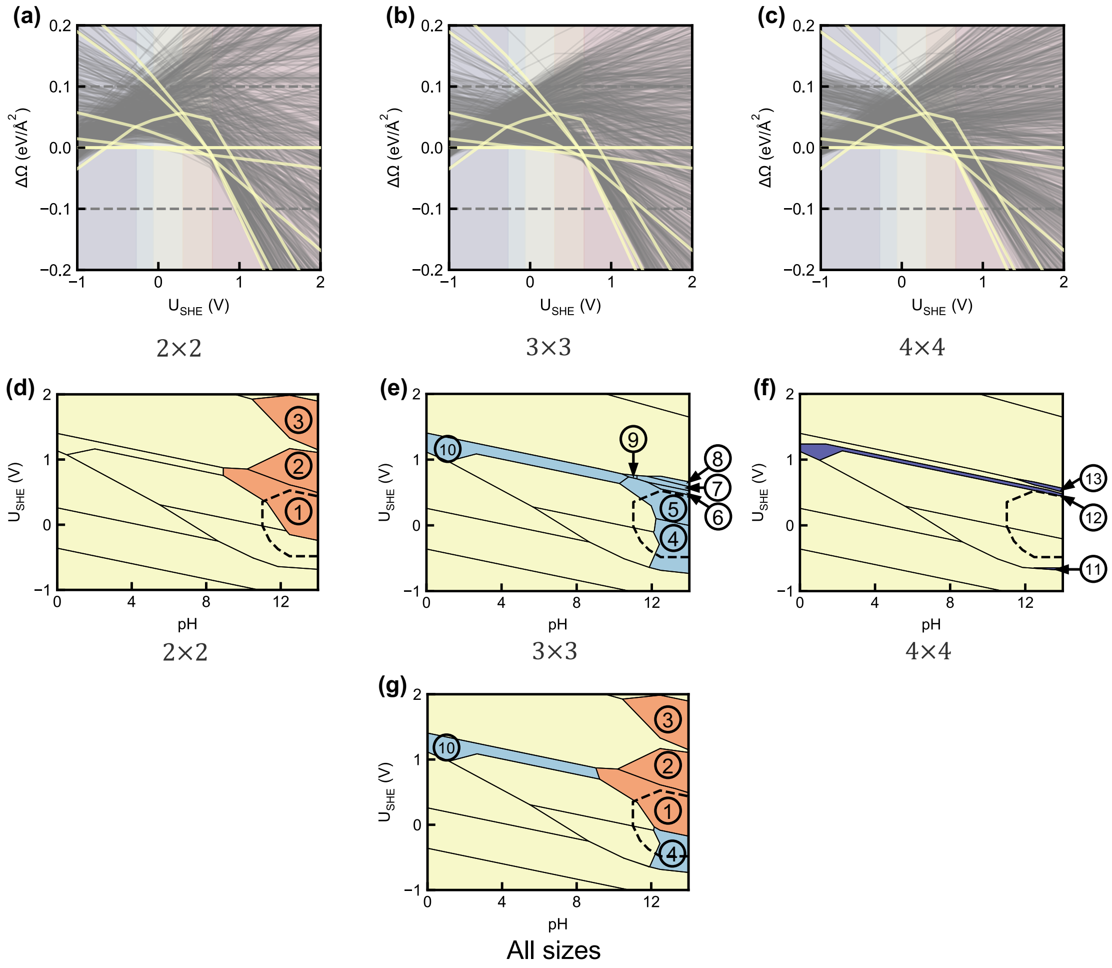

Figure S11: **Additional $LaMnO_3(001)$ energy analysis and surface Pourbaix diagrams at various supercell sizes with fine-tuned CHGNet energies.** (a-c) $\Delta\Omega_{\mathrm{surf}}(U_{\mathrm{SHE}}, \mathrm{pH})$ with respect to the pristine surface in $\mathrm{eV}/\mathrm{\AA}^2$ of handpicked and VSSR-MC sampled structures evaluated across U_{SHE} at fixed pH = 12. Handpicked structures are highlighted in yellow while sampled structures are grayed. Dashed lines are a guide for the eye. (d-g) $LaMnO_3(001)$ surface Pourbaix diagrams from fine-tuned NFF energies of (d) 2×2 structures, (e) 3×3 structures, (f) 4×4 structures, and (g) structures from all supercell sizes. Handpicked domains are in yellow, 2×2 sampled domains are in orange, 3×3 sampled domains are in light blue, and 4×4 sampled domains are in dark blue. Dashed lines enclose the $LaMnO_3(001)$ bulk stability region at 10^{-6} M dissolved species concentrations.

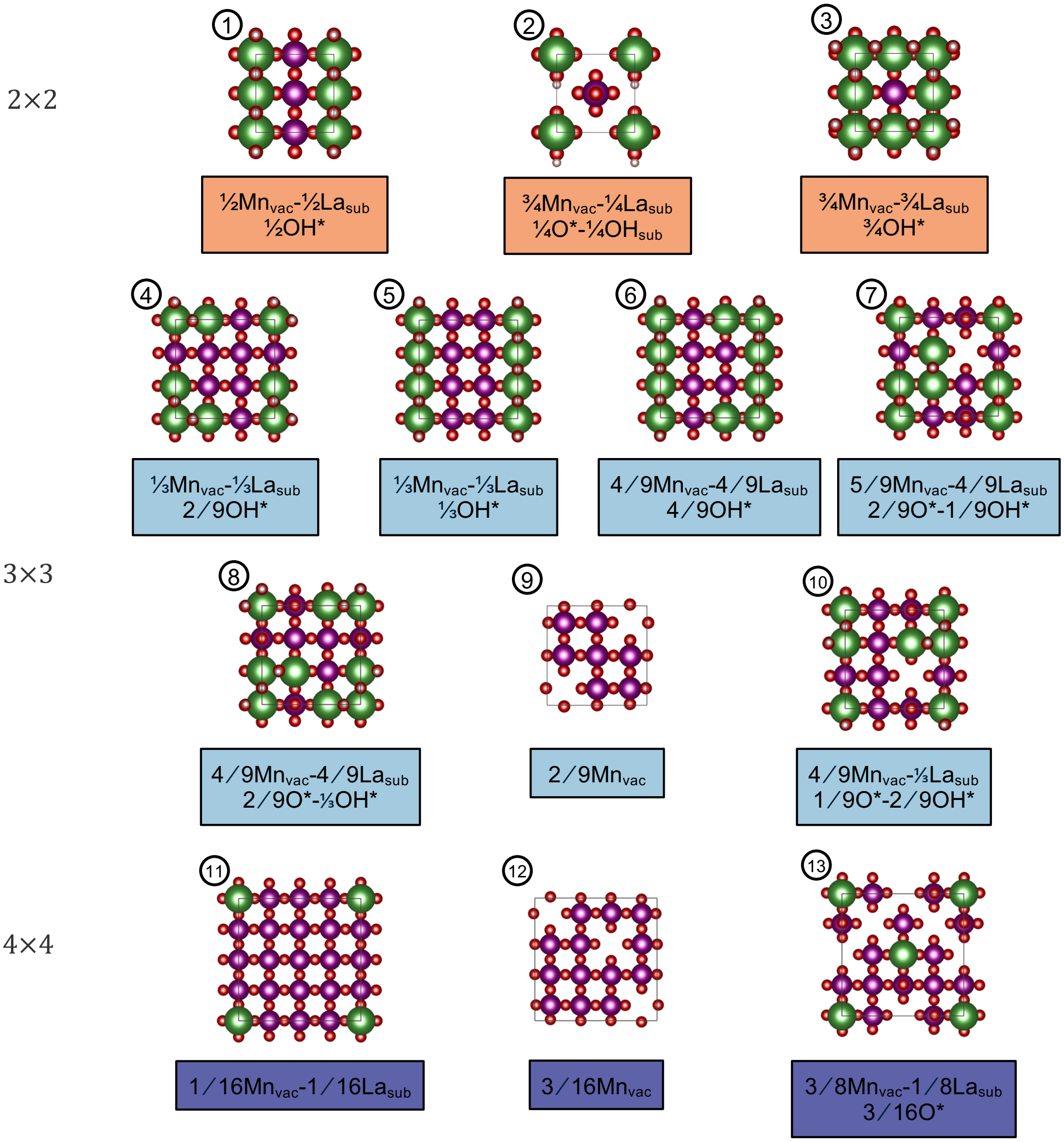

Figure S12: **Top-down view of new stable $LaMnO_3$(001) surfaces at all supercell sizes with fine-tuned CHGNet energies.** Numbers correspond to the phases labeled in Fig. S11. 2×2 surfaces are labeled in orange, 3×3 supercells are labeled in light blue, and 4×4 supercells are labeled in dark blue.

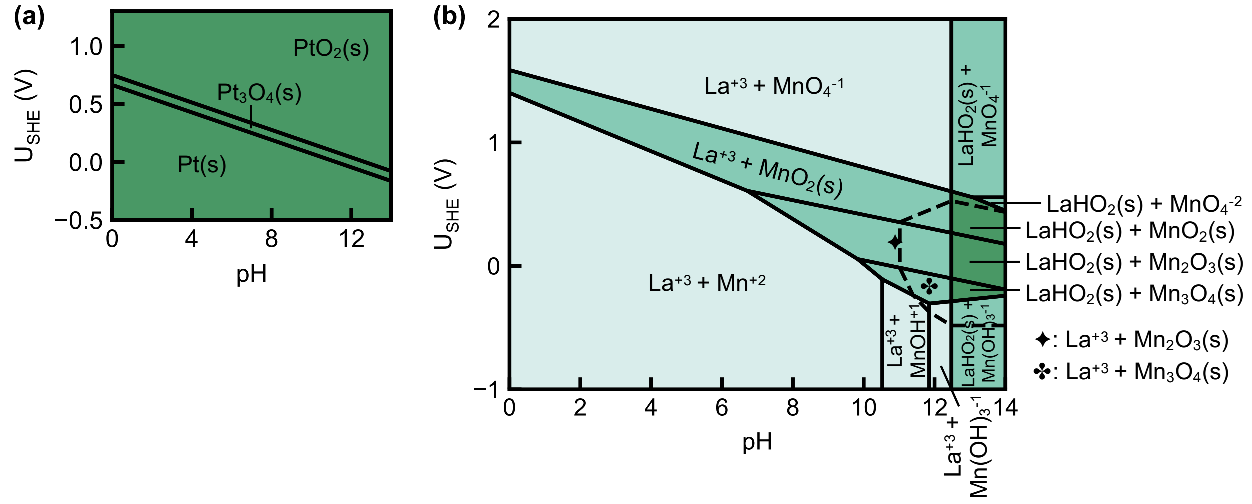

Figure S13: **Species Pourbaix diagrams of (a) Pt and (b) $LaMnO_3$ at 10^{-6} M dissolved species concentrations annotated with the stable solid or dissolved species at each combination of pH and U_{SHE}.** Solid species are in dark green, dissolved species are in pale blue, while mixed species are in teal. The bulk stability region for $LaMnO_3$ is encircled by dashed lines to reveal the underlying La and Mn species used in VSSR-MC sampling.

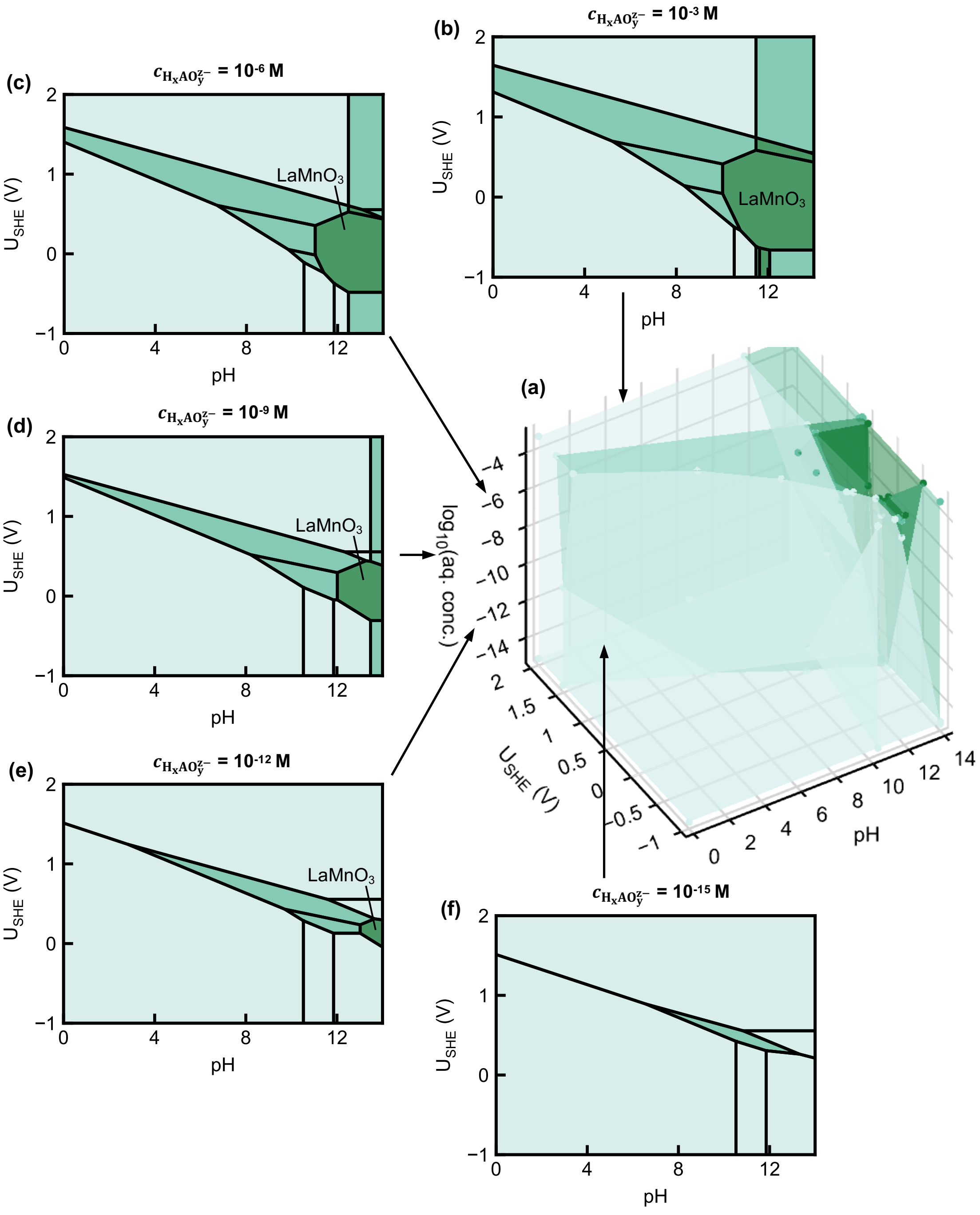

Figure S14: **pH-$U_{\mathbf{SHE}}$-$\log_{10} c_{\mathrm{H_xAO_y^{z-}}}$ 3D species Pourbaix diagram and pH-$U_{\mathbf{SHE}}$ 2D slices at fixed $\log_{10} c_{\mathrm{H_xAO_y^{z-}}}$ plotted with $LaMnO_3$ bulk stability region. Solid species are in dark green, dissolved species are in pale blue, while mixed species are in teal.** (a) 3D species Pourbaix diagram. 2D slices are extracted at $c_{\mathrm{H_xAO_y^{z-}}}$ = (b) 10^{-3} M, (c) 10^{-6} M, (d) 10^{-9} M, (e) 10^{-12} M, and (f) 10^{-15} M.

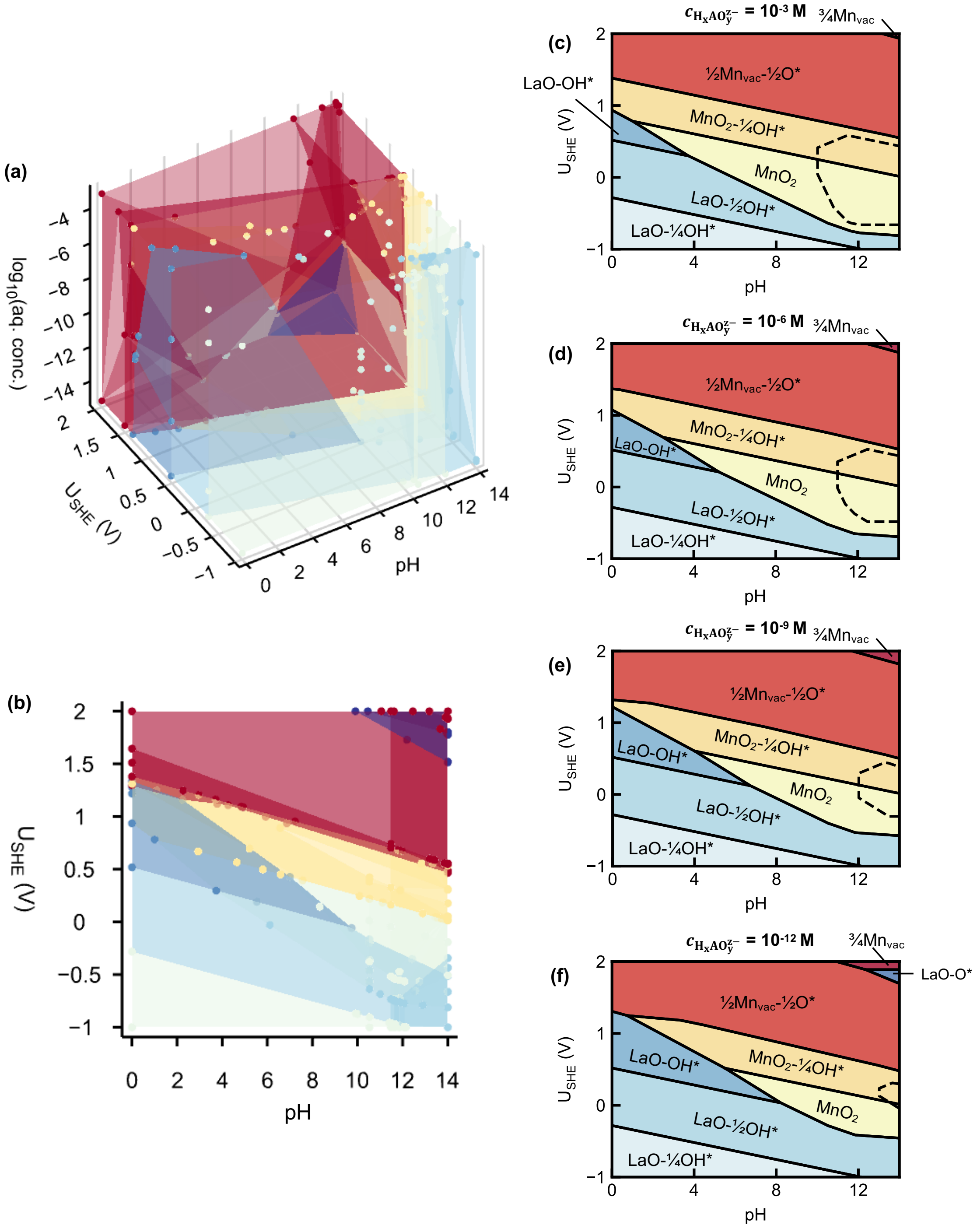

Figure S15: **pH-U_{SHE}-$\log_{10} c_{\mathrm{H_xAO_y^{z-}}}$ 3D surface Pourbaix diagram with literature surfaces and pH-U_{SHE} 2D slices at fixed $c_{\mathrm{H_xAO_y^{z-}}}$ plotted with $LaMnO_3$ bulk stability region enclosed by the dashed lines. Pristine surfaces are in light shades while more oxidized phases appear darker.** (a) 3D surface Pourbaix diagram. (b) 2D projection of surface Pourbaix diagram from high $c_{\mathrm{H_xAO_y^{z-}}}$. 2D slices are extracted at $c_{\mathrm{H_xAO_y^{z-}}}$ = (c) 10^{-3} M, (d) 10^{-6} M, (e) 10^{-9} M, and (f) 10^{-12} M.

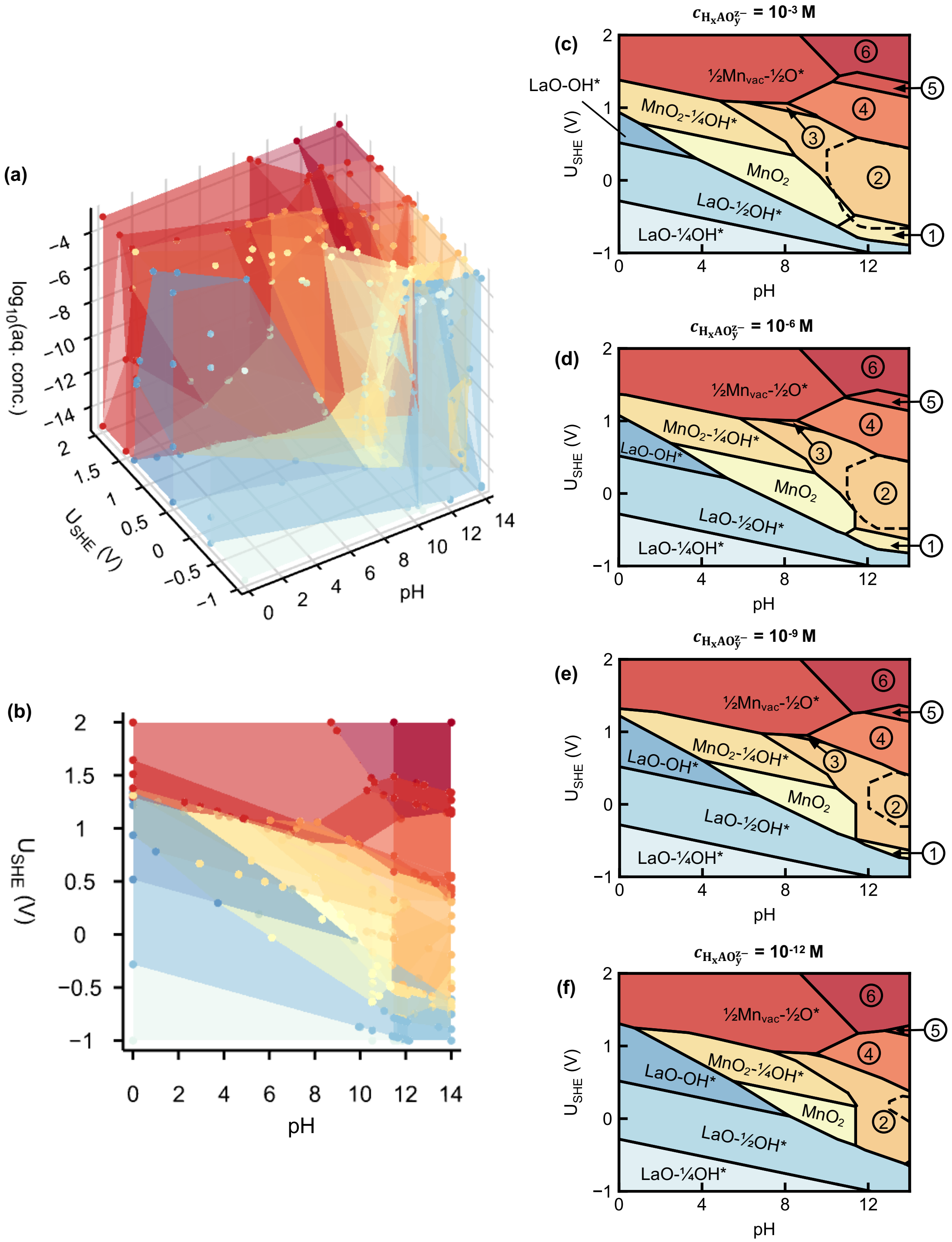

Figure S16: **pH-$U_{\mathbf{SHE}}$-$\log_{10} c_{H_xAO_y^{z-}}$ 3D surface Pourbaix diagram with literature and additional sampled surfaces, and pH-$U_{\mathbf{SHE}}$ 2D slices at fixed $c_{H_xAO_y^{z-}}$ plotted with $LaMnO_3$ bulk stability region enclosed by the dashed lines. Pristine surfaces are in light shades while more oxidized phases appear darker.** (a) 3D surface Pourbaix diagram. (b) 2D projection of surface Pourbaix diagram from high $c_{H_xAO_y^{z-}}$. 2D slices are extracted at $c_{H_xAO_y^{z-}}$ = (c) 10^{-3} M, (d) 10^{-6} M, (e) 10^{-9} M, and (f) 10^{-12} M. The numbered phases correspond to the sampled surfaces in Fig. 4(c).

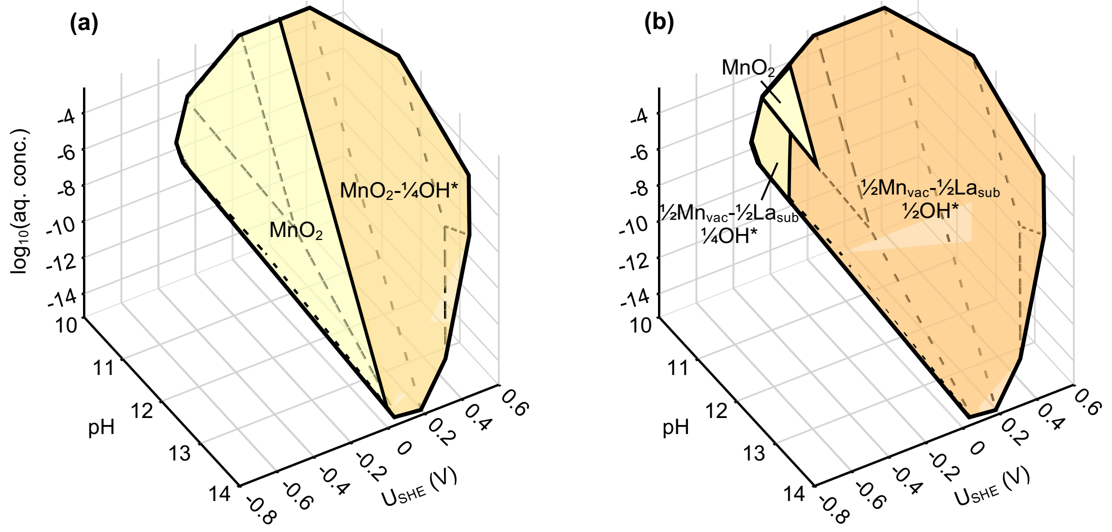

Figure S17: **3D perspectives of surface Pourbaix diagrams at thermodynamic equilibrium in the pH-$U_{\mathbf{SHE}}$-$\log_{10} c_{\mathrm{H_xAO_y^{z-}}}$ axes. More oxidized phases appear darker.** (a) With literature surfaces. (b) With literature and additional sampled surfaces.

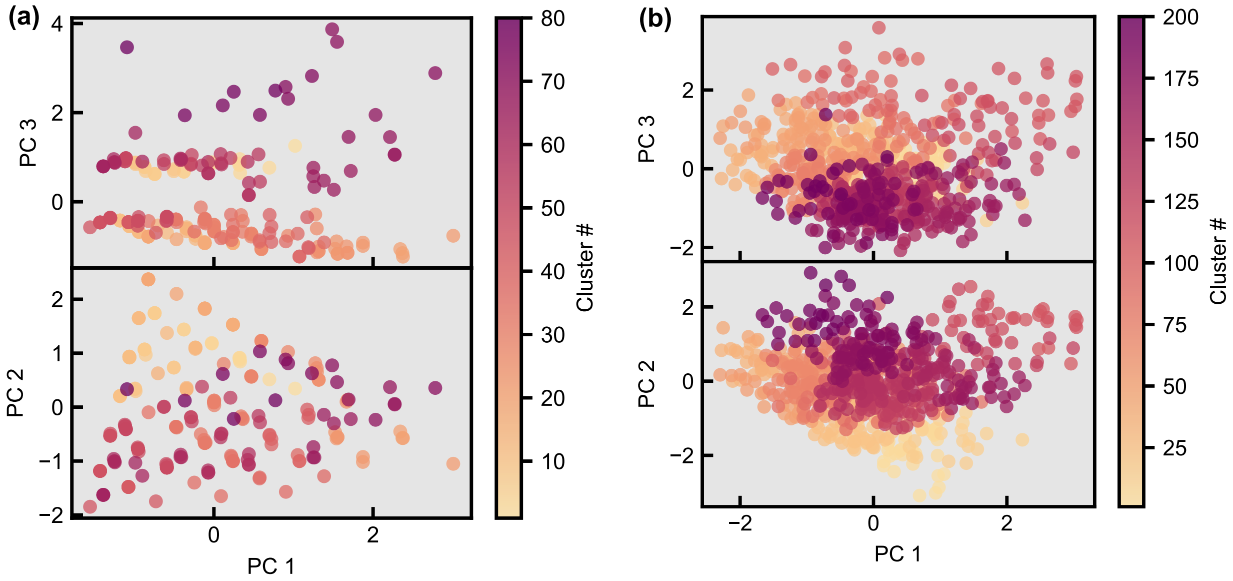

Figure S18: **Clustering of VSSR-MC structures using NFF embeddings visualized using the first 3 PCs.** (a) $(\sqrt{3} \times 3)$R30° Pt(111), (b) 2×2 $LaMnO_3$(001).

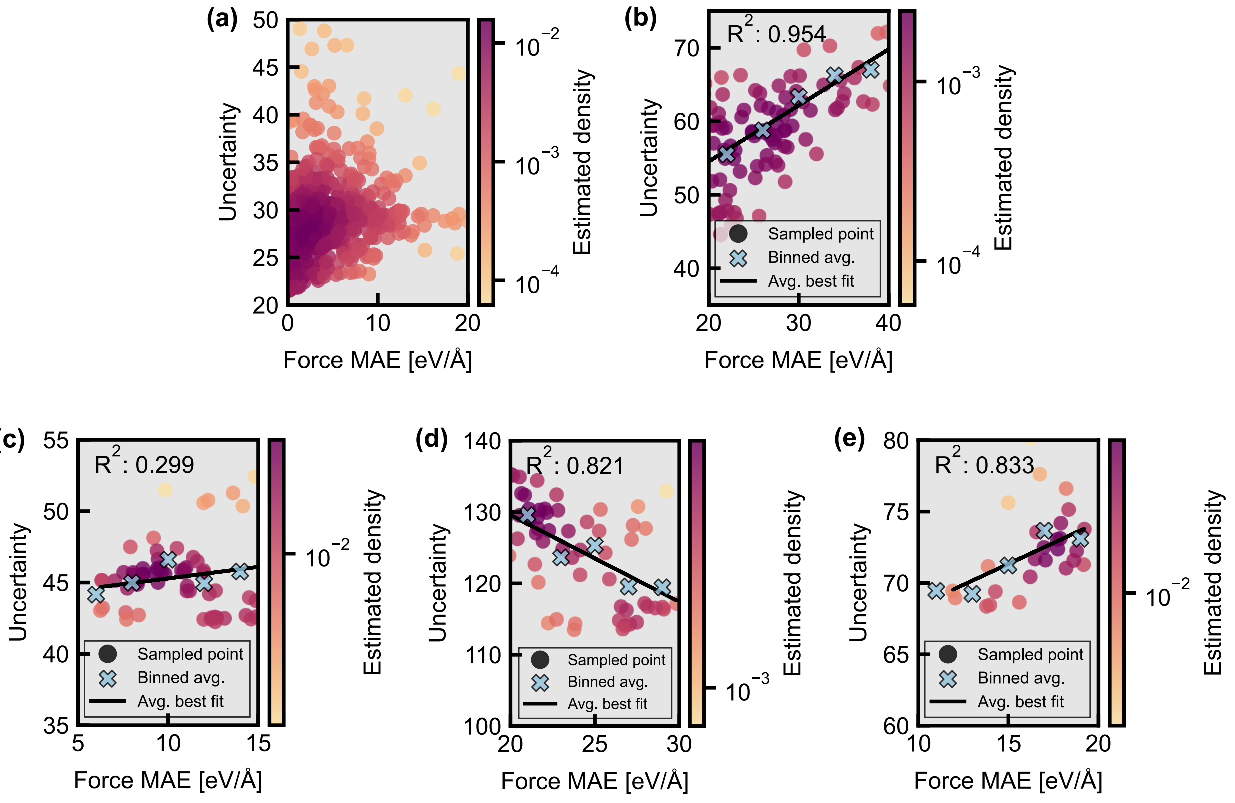

Figure S19: **GMM uncertainty calibration and result plots.** (a) Calibration with a subset of 5000 structures from MPtrj.[S5] The calibration performance is commensurate with previous work.[S6,S7] (b) 2×2 $LaMnO_3(001)$ fine-tuning dataset and (c-e) 2×2, 3×3, and $\sqrt{3} \times 3$ Pt(111) fine-tuning datasets. (b) and (e) showed good positive correlation between estimated uncertainty and force MAE. (c) showed a lower correlation while (d) showed a negative correlation, which could be due to both the relatively low-data regime and Pt(111) with O*/OH* being more out of distribution with respect to the MPtrj dataset compared with $LaMnO_3(001)$ surfaces.

Supplementary tables

Table S1: **Free energy of reaction for each Pt species at 298 K.**

A	$H_xAO_y^{Z-}$	$\Delta\Omega^{\ominus}_{A,SHE}$/eV	$\Delta\Omega^{\ominus}_{A,SHE} - 2.3N_{A,H^+}k_BT\text{pH} - N_{A,e}(eU_{SHE}) + k_BT\ln a_{H_xAO_y^{Z-}}$/eV
Pt	Pt(s)	0	0
	Pt_3O_4(s)	1.771	$1.771 - 0.158\text{pH} - 2.667eU_{SHE}$
	PtO_2(s)	2.770	$2.770 - 0.236\text{pH} - 4eU_{SHE}$
O	H_2O	−2.458	$-2.458 + 0.118\text{pH} + 2eU_{SHE}$
H	H^+	0	$-0.0591\text{pH} - eU_{SHE}$

Table S2: **Free energy of reaction for each $LaMnO_3$ species at 298 K.**

A	$H_xAO_y^{Z-}$	$\Delta\Omega^{\ominus}_{A,SHE}$/eV	$\Delta\Omega^{\ominus}_{A,SHE} - 2.3N_{A,H^+}k_BT\text{pH} - N_{A,e}(eU_{SHE}) + k_BT\ln a_{H_xAO_y^{Z-}}$/eV
Mn	Mn^{2+}	−2.316	$-2.316 - 2eU_{SHE} + 0.0257\ln a_{Mn^{2+}}$
	MnO(s)	−1.183	$-1.183 - 0.118\text{pH} - 2eU_{SHE}$
	MnO_2(s)	0.133	$0.133 - 0.236\text{pH} - 4eU_{SHE}$
	Mn_2O_3(s)	−0.872	$-0.872 - 0.177\text{pH} - 3eU_{SHE}$
	Mn_3O_4(s)	−1.084	$-1.084 - 0.158\text{pH} - 2.667eU_{SHE}$
	MnO_4^-	5.246	$5.246 - 0.472\text{pH} - 7eU_{SHE} + 0.0257\ln a_{MnO_4^-}$
	MnO_4^{2-}	4.691	$4.691 - 0.472\text{pH} - 6eU_{SHE} + 0.0257\ln a_{MnO_4^{2-}}$
	$MnOH^+$	−1.694	$-1.694 - 0.0591\text{pH} - 2eU_{SHE} + 0.0257\ln a_{MnOH^+}$
	$Mn(OH)_3^-$	−0.293	$-0.293 - 0.177\text{pH} - 2eU_{SHE} + 0.0257\ln a_{Mn(OH)_3{}^-}$
La	La^{3+}	−7.450	$-7.450 - 3eU_{SHE} + 0.0257\ln a_{La^{3+}}$
	$LaHO_2$(s)	−5.593	$-5.593 - 0.177\text{pH} - 3eU_{SHE}$
O	H_2O	−2.458	$-2.458 + 0.118\text{pH} + 2eU_{SHE}$
H	H^+	0	$-0.0591\text{pH} - eU_{SHE}$

Table S3: **Comparison of VSSR-MC with existing computational methods for electrochemical interface reconstruction.** Under "Algorithm", we have Gaussian process regression (GPR), gradient boosting regression (GBR), random forest regression (RFR), grand canonical Monte Carlo (GCMC), basin hopping (BH), persistent homology (PH), molecular dynamics (MD), simulated annealing (SA), and Gaussian approximation potential (GAP). Under "Best surrogate model performance", "F" refers to Force MAE while "E" refers to Energy MAE.

Reference	Algorithm	Samples across compositions?	Trains a surrogate energy model?	Best surrogate model performance	Adsorbates for most complicated run	Distinct compositions for each system	DFT calculations per composition
Ulissi *et al.*, 2016 [78]	Manual selection + GPR	**Automatic**	**Yes, GPR**	F: N/A<br>E: ~140 meV	3 species on IrO_2<br>**5 species on MoS_2**	IrO_2: <500 mean-field configs<br>MoS_2: <126 mean-field configs	IrO_2: ~20 relaxations total<br>MoS_2: ~35 relaxations total
Ghanekar *et al.*, 2022 [79]	SurfGraph + NFF	No	**Yes, NFF**	F: N/A<br>E: >=20 meV/ads	1 (NO* on Pt_3Sn & OH* on Pt)	Pt_3Sn: 6<br>Pt: 11	Pt_3Sn: ~58 DFT relaxations<br>Pt: ~127 DFT relaxations
Bang *et al.*, 2023 [80]	Random selection + NFF	No	**Yes, NFF**	E: 70 meV/ads (OH*)	2 (O*, OH* on Pt)	~ 100 (9 Pt isomorphs * 2 ads. types * 5 coverages)	~7 relaxations
Sharma *et al.*, 2024 [81]	Manual selection + classical ML (GBR, RFR)	No	**Yes, ML regressor**	E: 110 meV/ads (OH*)	3 (O*, OH*, OOH* on 28 transition metal clusters)	3 per transition metal cluster	1 relaxation
Qin *et al.*, 2024 [57]	GCMC BH	**Automatic**	No, DFT	-	3 (Ru, P, H on RuP_2)	RuP_2: estimated 30-80	~7–18 relaxations
Zheng *et al.*, 2025 [82]	PH-SA	No	**Yes, fine-tuned NFF**	F: 45 meV/Å (Pd)<br>**E: 1 meV/atom (Pd)**	3 (H* on Pd & O* on Pt slabs and clusters)	Pd: 10<br>Pt: 10	Pd: 50 relaxations<br>Pd: 6000 AIMD frames
Lee and Lee, 2025 [53]	MD, SA + GAP	No	**Yes, GPR**	F: 236 meV/Å<br>E: 21 meV/atom	3 (Bi, V, O on $BiVO_4$)	*p*(1x1) $BiVO_4$: 13	546 single point, 27 DFT relaxations
**Du *et al.*, 2025 [current work]**	VSSR-Pourbaix + NFF	**Automatic**	**Yes, fine-tuned NFF**	**F: 32 meV/Å (Pt)**<br>E: 4 meV/atom (Pt)	4 (La, Mn, O*, OH* on $LaMnO_3$)	Pt: 52<br>**$LaMnO_3$: 245**	Pt: 3 single point<br>**$LaMnO_3$: 0.5 single point**

References

(S1) Hansen, H. A.; Rossmeisl, J.; Nørskov, J. K. Surface Pourbaix diagrams and oxygen reduction activity of Pt, Ag and Ni(111) surfaces studied by DFT. *Physical Chemistry Chemical Physics* **2008**, *10*, 3722–3730, Publisher: The Royal Society of Chemistry.

(S2) Vinogradova, O.; Krishnamurthy, D.; Pande, V.; Viswanathan, V. Quantifying Confidence in DFT-Predicted Surface Pourbaix Diagrams of Transition-Metal Electrode–Electrolyte Interfaces. *Langmuir* **2018**, *34*, 12259–12269, Publisher: American Chemical Society.

(S3) Rong, X.; Kolpak, A. M. Ab Initio Approach for Prediction of Oxide Surface Structure, Stoichiometry, and Electrocatalytic Activity in Aqueous Solution. *The Journal of Physical Chemistry Letters* **2015**, *6*, 1785–1789.

(S4) Du, X. Data for: Accelerating and enhancing thermodynamic simulations of electrochemical interfaces. 2025; `https://doi.org/10.5281/zenodo.15066440`.

(S5) Deng, B. Materials Project Trajectory (MPtrj) Dataset. 2023; `https://figshare.com/articles/dataset/Materials_Project_Trjectory_MPtrj_Dataset/23713842/2`.

(S6) Zhu, A.; Batzner, S.; Musaelian, A.; Kozinsky, B. Fast uncertainty estimates in deep learning interatomic potentials. *The Journal of Chemical Physics* **2023**, *158*, 164111.

(S7) Tan, A. R.; Urata, S.; Goldman, S.; Dietschreit, J. C. B.; Gómez-Bombarelli, R. Single-model uncertainty quantification in neural network potentials does not consistently outperform model ensembles. *npj Computational Materials* **2023**, *9*, 1–11, Publisher: Nature Publishing Group.
