# Supplementary material for: Accelerating and Enhancing Thermodynamic Simulations of Electrochemical Interfaces
Source: ACS Cent Sci. 2025 Jul 21;11(9):1558–72. doi: 10.1021/acscentsci.5c00547 (PMC12464767; doi:10.1021/acscentsci.5c00547)
Supplement: Supplementary file 2 [file oc5c00547_si_002.pdf]

oc-2025-00547m.R1

Name: Peer Review Information for "Accelerating and enhancing thermodynamic simulations of electrochemical interfaces"

First Round of Reviewer Comments

Reviewer: 1

Comments to the Author

This is an interesting paper by Gomez-Bombarelli and coworkers on the application of the Virtual Surface Site Relaxation-Monte Carlo (VSSR-MC) algorithm for electrochemistry, more specifically, electrocatalysis. While the authors developed this method in a Nature Comp Sci article in 2023, here it is - to the best of my knowledge - applied for the first time to solid/ liquid interfaces relevant to energy conversion and storage. Therefore, I am convinced that this paper falls into the scope of ACS Central Science in terms of novelty and relevance.

My main criticism is that this paper is written in a way that the reader must have read the previous work in Nat Comp Sci to fully grasp the authors' procedure. I would appreciate if the authors could provide additional information on the workflow - without inflating the size of the manuscript - because this makes the paper accessible to a broader audience.

Scientifically, my main concern is the treatment of the cHxAOz−species.

A) How do the authors calculate a charged HxAOz−species with sufficient accuracy using plane-wave DFT in the realm of VASP?

B) The authors refer to the "most stable aqueous species HxAOz−species", but I could not see that these species are clearly stated. In other words, what are the reference states for HxAOz− in case of i) Pt, ii) LaMnO3?

C) The authors claim that they look at the concentration dependence of HxAOz− - does this really matter? In the end, the "concentration dependence" is a simple kBT x ln(c) term, which is on the order of 0.059 eV x log (c); if c varies by one order of magnitude, the effect is about 0.06 eV, which is below the error bars of DFT. Therefore, I am wondering if this claim

is justified. Why not using a fixed concentration of HxAOz− ? Or comparing c = 1 mol/L and c = 10^-6 mol/L?

D) It does not get clear to me how the authors arrive at the conclusion that the Pourbaix diagram for Pt(111) in figure 2f is "correct". What is the reference work that the authors use?

E) While this statement mainly holds for metals rather than for metal oxides, grand canonical (GC) DFT might be needed to determine accurate dG values and thus Pourbaix diagrams in case of Pt. Can the proposed framework be extended toward the GC-DFT approach? Some outline might be provided.

Reviewer: 2

Comments to the Author

Comments on 'Accelerating and enhancing thermodynamic simulations of electrochemical interfaces'

In their manuscript Xiaochen Du and colleagues present an adaptation of Virtual Surface Site RelaxationMonte Carlo (VSSR-MC) for thermodynamic sampling of surfaces under aqueous condition. The subsequent combination of MLFFs and VSSR-MC allows them to accelerate the creation of pourbaix diagrams. The authors first used Pt oxidation as a simple system to benchmark a pretrained foundational model and a fine-tune foundational model with regard to DFT and show that VSSR-MC can be used to fine-tune their MLFF. They show again that the same holds for a more complex system (LaMnO3). For LaMnO3 the authors show that VSSR-MC can be used for enhanced sampling leading to plenty unique surface configurations, including a couple of configurations lower in energy than the ones cited from literature, that were sampled through human intuition. In addition, they highlight the window of thermodynamic stability of their underlying bulk phase with varying ion concentrations.

The work presented is of high scientific standard, understandable, and well written. The paper provides a state-of-the-art workflow for efficiently computing surface Pourbaix diagrams. However, there are several shortcomings that require a major revision. The manuscript focuses mainly on the methodology without showing how the approach outperforms other surface sampling schemes. A shift towards the application in combination with a larger model size (4x4 or 6x6) would show that this approach can deal with surfaces that are impossible to sample manually through human intuition and thus

make the paper significantly more interesting. I would recommend that the manuscript addresses the following comments to be suitable for publication in ACS central science.

Major comments:

- This work uses a slightly adapted version of previously published code in combination with an MLFF. How does the presented approach outperform other surface sampling approaches as presented by Robert B. Wexler (https://doi.org/10.1021/acs.jpcc.8b11093) and Yonghyuk Lee (https: //doi.org/10.1021/acs.jpcc.3c04049)?
- The more complex LaMcO3 runs took 70 min on a 2x2 supercell. The manuscript would benefit a lot from using a larger supercell and inclusion of how the needed compute time scales with system size.
- The long discussion and benchmark of pre-trained vs. fine-tuned foundational model does not really contribute to accelerated creation of pourbaix diagrams. Taking into account that the MACE-MP0 paper (https://arxiv.org/abs/2401.00096v2) already discourages taking the pre-trained model from scratch, it increases readability if the section (or at least major parts) **Validation modified VSSR-MC and fine-tuning** (pages 7-11) are shifted in the SI.

Minor comments:

- The calculations or sources from which the $\Delta\Omega_{\mathrm{surf}}$ values originate are missing. A table with the values used for $\Delta\Omega_{\mathrm{surf}}$ and μ_{A} would greatly benefit reproducibility.
- The definition of a **sweep** is not completely clear.
- The VSSR-MC is performed semigrand-canonical, which puts limitations on the covered composition space: Can you state how you cover the necessary composition space for your finite-sized cell?
- The geometry optimizations are limited to 20 steps (page 20). Why is the step limit set so low? How often was that limit reached? • Why is the surface grand potential normalized by the unit cell and not the surface area as commonly done in *ab-initio* thermodynamics?

Author's Response to Peer Review Comments:

oc-2025-00547m: Accelerating and enhancing thermodynamic simulations of electrochemical interfaces

Reviewer: 1

Comments:
This is an interesting paper by Gomez-Bombarelli and coworkers on the application of the Virtual Surface Site Relaxation-Monte Carlo (VSSR-MC) algorithm for electrochemistry, more specifically, electrocatalysis. While the authors developed this method in a Nature Comp Sci article in 2023, here it is - to the best of my knowledge - applied for the first time to solid/ liquid interfaces relevant to energy conversion and storage. Therefore, I am convinced that this paper falls into the scope of ACS Central Science in terms of novelty and relevance.
My main criticism is that this paper is written in a way that the reader must have read the previous work in Nat Comp Sci to fully grasp the authors' procedure. I would appreciate if the authors could provide additional information on the workflow - without inflating the size of the manuscript - because this makes the paper accessible to a broader audience.
We sincerely thank the Reviewer for their supportive comments and for recognizing the novelty and relevance of this work. We also appreciate the suggestion to improve accessibility for readers unfamiliar with our prior publication. In response, we have added a concise overview of the original VSSR-MC framework in the **Computational workflow and method development** section. We have also clarified how this work extends VSSR-MC to solid-liquid electrochemical interfaces and distinguished the new methodological contributions from our earlier work. These additions aim to make the workflow more self-contained while keeping the manuscript focused and concise.

Scientifically, my main concern is the treatment of the cHxAOz−species.
A) How do the authors calculate a charged HxAOz−species with sufficient accuracy using plane-wave DFT in the realm of VASP?
We do not directly calculate the energies of charged aqueous species using plane-wave DFT. Instead, we use experimentally tabulated energies from the Aqueous Ion Reference Data hosted on the Materials Project. These values are then corrected to be thermodynamically consistent with the DFT energies for solid-phase species, which were also obtained from the Materials Project. We have revised the **Methods: Species Pourbaix diagram construction** and **Methods: Surface Pourbaix grand potential** sections for clarity.

B) The authors refer to the "most stable aqueous species HxAOz−species", but I could not see that these species are clearly stated. In other words, what are the reference states for HxAOz− in case of i) Pt, ii) LaMnO3?
The reference states correspond to the most stable aqueous species identified in the species Pourbaix diagrams (**Fig. S13**) under the specified electrochemical conditions. Details are provided in **Methods: Species Pourbaix diagram construction** and **Methods: Surface Pourbaix grand potential** sections. We have also revised **Computational workflow and method development** to clarify this point.

C) The authors claim that they look at the concentration dependence of HxAOz− - does this really matter? In the end, the "concentration dependence" is a simple kBT x ln(c) term, which is on the order of 0.059 eV x log (c); if c varies by one order of magnitude, the effect is about 0.06 eV, which is below the error bars of DFT. Therefore, I am wondering if this claim is justified. Why not using a fixed concentration of HxAOz− ? Or comparing c = 1 mol/L and c = 10^-6 mol/L?
Our formulation is agnostic to the level of theory used to obtain the energies for the reference species and surfaces. In this work, the PBE functional was used for surfaces and solid-phase reference species, which has an estimated accuracy of ~0.10 eV/atom. If higher-level methods or experimental data were used instead, the energetic precision could be further improved. We have clarified this point in the **Computational workflow and method development** section.
As the reviewer noted, the concentration dependence introduces a modest shift of ~0.06 eV/atom per order of magnitude. However, across the wide concentration range we studied (10^{-15} to 10^{-3} mol/L), this amounts to **a total variation of ~0.72 eV/atom**, which is **sufficient to alter the identity of the most stable species and surface domains**, as demonstrated in **Figs. 5 and S17**.
The purpose of including this concentration dependence is not to resolve small energetic differences but to reflect the dynamic coupling between surface phases and realistic electrolyte conditions, as we state in the **Computational workflow and method development** section. A fixed concentration of 10^{-6} mol/L is often overly idealized while 1 mol/L might be too concentrated for ordinary electrocatalyst operational conditions [R1].

Reference:
[R1] Kan, K.; Guevarra, D.; Zhou, L.; Jones, R. J. R.; Lai, Y.; Richter, M.; Gregoire, J. M. Accelerated Characterization of Electrode-Electrolyte Equilibration. ChemCatChem 2024, 16 (6), e202301300.

D) It does not get clear to me how the authors arrive at the conclusion that the Pourbaix diagram for Pt(111) in figure 2f is "correct". What is the reference work that the authors use?
Fig. 2(f) shows the surface Pourbaix diagram predicted using the fine-tuned NFF and is in close agreement with **Fig. 2(d)**, which was constructed using DFT energies for known Pt(111) surface structures. These structures were sourced from prior literature [R2-3], as described in the **Assessing pre-trained and fine-tuned NFF performance** section.
While the exact phase boundaries may vary with the choice of DFT functional or software, our DFT-based diagram in Fig. 2(d) serves as the reference for assessing the accuracy of the fine-tuned NFF. No new lower-energy phases were found in the pre-trained NFF sampling (see Fig. S9), so the literature-derived structures were deemed sufficient.

References:
[R2] Hansen, H. A.; Rossmeisl, J.; Nørskov, J. K. Surface Pourbaix Diagrams and Oxygen Reduction Activity of Pt, Ag and Ni(111) Surfaces Studied by DFT. *Phys. Chem. Chem. Phys.* **2008**, *10* (25), 3722–3730.
[R3] Vinogradova, O.; Krishnamurthy, D.; Pande, V.; Viswanathan, V. Quantifying Confidence in DFT-Predicted Surface Pourbaix Diagrams of Transition-Metal Electrode–Electrolyte Interfaces. *Langmuir* **2018**, *34* (41), 12259–12269.

E) While this statement mainly holds for metals rather than for metal oxides, grand canonical (GC) DFT might be needed to

determine accurate dG values and thus Pourbaix diagrams in case of Pt. Can the proposed framework be extended toward the GC-DFT approach? Some outline might be provided.

Yes, the proposed framework can be extended to incorporate the electronically grand-canonical (GC) ensemble required for constant-potential simulations. Since VSSR-MC is agnostic to the underlying energy model, it is compatible with surrogate models trained on GC-DFT data. For instance, a machine-learned force field could be constructed to predict charge-dependent energies by taking the electrode potential, atomic species, and positions as inputs, enabling direct estimation of grand-canonical surface energies and surface charges. Such approaches are already being explored, as in Ref. [R4].

We briefly alluded to this in the **Discussion** section, where we noted that the computational hydrogen electrode (CHE) framework used in this work neglects the role of electrode charging and the electric double layer. Future extensions of VSSR-MC could incorporate solvation and grand-canonical effects to better capture these interfacial phenomena. We have since included additional discussion to the **Discussion** section.

Reference:

[R4] Chen, X.; El Khatib, M.; Lindgren, P.; Willard, A.; Medford, A. J.; Peterson, A. A. Atomistic Learning in the Electronically Grand-Canonical Ensemble. *npj Comput Mater* **2023**, *9* (1), 1–9.

Reviewer: 2

In their manuscript Xiaochen Du and colleagues present an adaptation of Virtual Surface Site Relaxation- Monte Carlo (VSSR-MC) for thermodynamic sampling of surfaces under aqueous condition. The subsequent combination of MLFFs and VSSR-MC allows them to accelerate the creation of pourbaix diagrams. The authors first used Pt oxidation as a simple system to benchmark a pretrained foundational model and a fine-tune foundational model with regard to DFT and show that VSSR-MC can be used to fine-tune their MLFF. They show again that the same holds for a more complex system (LaMnO3). For LaMnO3 the authors show that VSSR-MC can be used for enhanced sampling leading to plenty unique surface configurations, including a couple of configurations lower in energy than the ones cited from literature, that were sampled through human intuition. In addition, they highlight the window of thermodynamic stability of their underlying bulk phase with varying ion concentrations.
The work presented is of high scientific standard, understandable, and well written. The paper provides a state-of-the-art workflow for efficiently computing surface Pourbaix diagrams. However, there are several shortcomings that require a major revision. The manuscript focuses mainly on the methodology without showing how the approach outperforms other surface sampling schemes. A shift towards the application in combination with a larger model size (4x4 or 6x6) would show that this approach can deal with surfaces that are impossible to sample manually through human intuition and thus make the paper significantly more interesting. I would recommend that the manuscript addresses the following comments to be suitable for publication in ACS central science.
We thank the Reviewer for their thoughtful summary and constructive feedback. In response to the request for stronger benchmarking and demonstration of scalability, we have significantly expanded our analysis. Specifically, we now include additional results using 3×3 and 4×4 supercells for $LaMnO_3$(001), demonstrating that VSSR-MC not only scales to larger, symmetry-breaking supercells but also uncovers new surface phases that are unlikely to be identified through human intuition. We also present a direct comparison to prior surface sampling approaches and clarify how VSSR-MC surpasses prior methods in scope, automation, and computational efficiency. These revisions address the Reviewer's key concerns and further highlight the versatility and impact of our approach. Detailed responses and supporting data are provided below.

Major comments:

- This work uses a slightly adapted version of previously published code in combination with an MLFF. How does the presented approach outperform other surface sampling approaches as presented by Robert B. Wexler (https://doi.org/10.1021/acs.jpcc.8b11093) and Yonghyuk Lee (https: //doi.org/10.1021/acs.jpcc.3c04049)?

In our **original VSSR-MC work, Supplementary Data 1** [R5], we listed a comprehensive comparison of VSSR-MC with previous state-of-the-art sampling/optimization methods for **surfaces in contact with gas/vacuum**, demonstrating the automation, sampling breadth, and DFT efficiency of our method. We reproduce that table over here as a subset of **Table R1** for the Reviewer's convenience and have updated the **Computational workflow and method development** section to guide readers to this table. Please refer to the original VSSR-MC work for full citations. Briefly, compared with Wexler *et al.* [R6], we are both effectively doing grand-canonical sampling across compositions and configurations, i.e., we vary not only the adsorbate positions but also the surface stoichiometry and net atomic content. However, Wexler *et al.* demonstrated their method on Ag_XO_y, an arguably simpler surface and uses expensive DFT calculation at each MC step. Meanwhile, we are backed with an NFF and sample a three-element oxide, $SrTiO_3$.

Our **original VSSR-MC** work was a concurrent work with Lee *et al.* [R7], and thus did not include their work in our initial comparison. While Lee *et al.* uses an ML force field, they must manually specify the composition for each simulated annealing run and cannot sample across compositions. They also tested on an easier system, IrO_2, and require a lot more DFT calculations per composition but achieving a lower accuracy than VSSR-MC. Please see the highlighted row in the updated comparison table below (**Table R1**). In essence, **our original VSSR-MC method remains state-of-the-art for sampling surfaces exposed to gas/vacuum**, combining general-purpose applicability across large compositional spaces with low per-composition DFT cost.

**Reference**	**Algorithm**	**Samples across compositions?**	**Trains a surrogate energy model?**	**Best surrogate model performance**	**Elements for most complicated run**	**Distinct compositions for each system**	**DFT calculations per composition**
Bisbo and Hammer	GOFEE (EA + GPR)	No	**Yes, GPR**	Not specified	3 (C and O on Ir)	<=12	>200 single point
Merte *et al.*	GOFEE	No	**Yes, GPR**	Not specified	3 (O on Pt_3Sn)	16	>200 single point
Timmermann *et al.*	MD, SA + GAP	No	**Yes, GAP**	E: 25 meV/atom	2 (IrO_2, RuO_2)	20-30	>5 relaxations, ~>50 single point
Rønne *et al.*	AGOX (BH + GAP)	No	**Yes, GAP**	E: 103.1 meV/atom	2 (Ag_xO_y)	12	>16 single point
Han *et al.*	MAGUS (EA)	Manually specified	No, DFT/pretrained FF	-	2 (SnO_2, SiC)	<10	>10 relaxations
Meldgaard *et al.*	ASLA (RL + DFT)	No	No, DFT	-	2 (TiO_2, SnO_2)	1	1000 single point
Wang *et al.*	USPEX (EA + DFT)	**Automatic**	No, DFT	-	2 (TiO_2)	Not specified	1800 total relaxations
Schusteritsch and Pickard	AIRSS (RSS + DFT)	Manually specified	No, DFT	-	3 ($SrTiO_3$)	5	-
Bauer *et al.*	BH and EA	No	No, classical FF	-	1 (Si)	1	-
Wexler *et al.* [R6]	GCMC BH	**Automatic**	No, DFT	-	2 (Ag_xO_y)	Not specified	>6000 total relaxations
Hess and Yildiz	MCMC + SA	No	No, electrostatic forces and DFT	-	**4 ($La_{0.75}Sr_{0.25}$ MnO_3)**	1	450 total relaxations
Wanzenböck *et al.*	CMA-ES + NFF	No	**Yes, NFF**	**F: 77.01 meV/Å<br>E: 0.836 meV/atom**	3 ($SrTiO_3$)	1 (training)	3000 single point
Xu *et al.*	GCMC + NFF	**Automatic**	**Yes, NFF**	F: 230 meV/Å<br>E: 6 meV/atom	2 (Pt_xO_y)	95 (training)	552 single point
Lee *et al.* [R7]	MD, SA + GAP	No	**Yes, GAP**	F: >100 meV/Å<br>E: ~8 meV/atom	2 (RuO_2)	*c*(2x2)-(100): 5<br>(1x1)-(410): 18	*c*(2x2)-(100): 261 single point<br>(1x1)-(410): 114 single point
**Du *et al.*, 2023 [R5]**	VSSR-MC + NFF	**Automatic**	**Yes, NFF**	F: 100 meV/Å<br>E: 5.18 meV/atom	3 ($SrTiO_3$)	**683**	**<8 single point**

Table R1. Comparison of VSSR-MC with existing computational methods for gas/vacuum surface reconstruction. Under "Algorithm", we have evolutionary algorithms (EA), Gaussian process regression (GPR), molecular dynamics (MD), simulated annealing (SA), Gaussian approximation potential (GAP), basin hopping (BH), reinforcement learning (RL), random structure search

(RSS), grand canonical Monte Carlo (GCMC), Markov-chain Monte Carlo (MCMC), and covariance matrix adaptation evolution strategy (CMA-ES). Under "Best surrogate model performance", "F" refers to Force MAE while "E" refers to Energy MAE.

Our modified VSSR-MC (henceforth denoted as **VSSR-Pourbaix**) in the **current work**, as mentioned in the main text, **further improves on our original VSSR-MC** with multicomponent adsorbates (multi-atom adsorbates such as HO* and H2O*) and uses a pre-trained NFF instead of training an NFF from scratch. In **Table R2** (now added to the SI as **Table S3**), we compare **VSSR-Pourbaix** to current literature methods focused on **exploring aqueous electrochemical interfaces**, including one from Yonghyuk Lee [R8]. These methods, as we cite in the main text, either "rely on costly *ab initio* grand canonical Monte Carlo (GCMC) sampling" or "examine reconstruction only within a narrow set of configurations—permitting select adsorbate and metal surface combinations but disallowing general surface dissolution and re-deposition—thus leaving the broader compositional space largely unexplored."

From **Table R2**, we see that **VSSR-Pourbaix** automatically samples across **245 compositions** for a **3-element perovskite**, many more than other competing algorithms, which do not perform sampling across compositions. Ulissi *et al.* was the only reference using an ML energy surrogate that automatically samples across adsorbates, still not allowing for general dissolution and redeposition of metal atoms. Additionally, their mean-field approximation used for computational efficiency sacrifices considerable accuracy. Amongst all these methods, **VSSR-Pourbaix** was able fine-tune an NFF with **arguably the lowest number of DFT calculations to performance ratio**, requiring **0.5 single point calculation per composition** for the complex $LaMnO_3$, while all other methods required DFT relaxations that take multiple steps each. In summary, **VSSR-Pourbaix** achieves **finer accuracy** of surface-phase equilibria with **dramatically fewer DFT calculations per composition** (≤ 1) compared to prior work, while **expanding the sampling scope to realistic multi-adsorbate aqueous electrochemical environments**. We have updated the **Discussion** section to highlight **Table S3**.

Reference	Algorithm	Samples across compositions?	Trains a surrogate energy model?	Best surrogate model performance	Adsorbates for most complicated run	Distinct compositions for each system	DFT calculations per composition
Ulissi *et al.*, 2016 [R9]	Manual selection + GPR	**Automatic**	**Yes, GPR**	F: N/A<br>E: ~140 meV	3 species on IrO_2<br>**5 species on MoS_2**	IrO_2: <500 mean-field configs<br>MoS_2: <126 mean-field configs	IrO_2: ~20 relaxations total<br>MoS_2: ~35 relaxations total
Ghanekar *et al.*, 2022 [R10]	SurfGraph + NFF	No	**Yes, NFF**	F: N/A<br>E: >=20 meV/ads	1 (NO* on Pt_3Sn & OH* on Pt)	Pt_3Sn: 6<br>Pt: 11	Pt_3Sn: ~58 DFT relaxations<br>Pt: ~127 DFT relaxations
Bang *et al.*, 2023 [R11]	Random selection + NFF	No	**Yes, NFF**	E: 70 meV/ads (OH*)	2 (O*, OH* on Pt)	~ 100 (9 Pt isomorphs * 2 ads. types * 5 coverages)	~7 relaxations
Sharma *et al.*, 2024 [R12]	Manual selection + classical ML (GBR, RFR)	No	**Yes, ML regressor**	E: 110 meV/ads (OH*)	3 (O*, OH*, OOH* on 28 transition metal clusters)	3 per transition metal cluster	1 relaxation
Qin *et al.*, 2024 [R13]	GCMC BH	**Automatic**	No, DFT	-	3 (Ru, P, H on RuP_2)	RuP_2: estimated 30-80	~7–18 relaxations
Zheng *et al.*, 2025 [R14]	PH-SA	No	**Yes, fine-tuned NFF**	F: 45 meV/Å (Pd)<br>**E: 1 meV/atom (Pd)**	3 (H* on Pd & O* on Pt slabs and clusters)	Pd: 10<br>Pt: 10	Pd: 50 relaxations<br>Pd: 6000 AIMD frames
Lee and Lee, 2025 [R8]	MD, SA + GAP	No	**Yes, GPR**	F: 236 meV/Å<br>E: 21 meV/atom	3 (Bi, V, O on $BiVO_4$)	*p*(1x1) $BiVO_4$: 13	546 single point, 27 DFT relaxations
**Du *et al.*, 2025 [current work]**	VSSR-Pourbaix + NFF	**Automatic**	**Yes, fine-tuned NFF**	**F: 32 meV/Å (Pt)**<br>E: 4 meV/atom (Pt)	4 (La, Mn, O*, OH* on $LaMnO_3$)	Pt: 52<br>**$LaMnO_3$: 245**	Pt: 3 single point<br>**$LaMnO_3$: 0.5 single point**

Table R2. Comparison of VSSR-Pourbaix with existing computational methods for electrochemical interface reconstruction. Under "Algorithm", we have Gaussian process regression (GPR), gradient boosting regression (GBR), random forest regression (RFR), grand canonical Monte Carlo (GCMC), basin hopping (BH), persistent homology (PH), molecular dynamics (MD), simulated annealing (SA), and Gaussian approximation potential (GAP). Under "Best surrogate model performance", "F" refers to Force MAE while "E" refers to Energy MAE.

References:

[R5] Du, X.; Damewood, J. K.; Lunger, J. R.; Millan, R.; Yildiz, B.; Li, L.; Gómez-Bombarelli, R. Machine-Learning-Accelerated Simulations to Enable Automatic Surface Reconstruction. *Nat Comput Sci* **2023**, *3* (12), 1034–1044.

[R6] Wexler, R. B.; Qiu, T.; Rappe, A. M. Automatic Prediction of Surface Phase Diagrams Using Ab Initio Grand Canonical Monte Carlo. *J. Phys. Chem. C* **2019**, *123* (4), 2321–2328.

[R7] Lee, Y.; Timmermann, J.; Panosetti, C.; Scheurer, C.; Reuter, K. Staged Training of Machine-Learning Potentials from Small to Large Surface Unit Cells: Efficient Global Structure Determination of the RuO2(100)-c(2 × 2) Reconstruction and (410) Vicinal. *J. Phys. Chem. C* **2023**, *127* (35), 17599–17608.

[R8] Lee, Y.; Lee, T. Machine-Learning-Accelerated Surface Exploration of Reconstructed BiVO4(010) and Characterization of Their Aqueous Interfaces. *J. Am. Chem. Soc.* **2025**, *147* (9), 7799–7808.

[R9] Ulissi, Z. W.; Singh, A. R.; Tsai, C.; Nørskov, J. K. Automated Discovery and Construction of Surface Phase Diagrams Using Machine Learning. *J. Phys. Chem. Lett.* **2016**, *7* (19), 3931–3935.

[R10] Ghanekar, P. G.; Deshpande, S.; Greeley, J. Adsorbate Chemical Environment-Based Machine Learning Framework for Heterogeneous Catalysis. *Nat Commun* **2022**, *13* (1), 5788.

[R11] Bang, K.; Hong, D.; Park, Y.; Kim, D.; Han, S. S.; Lee, H. M. Machine Learning-Enabled Exploration of the Electrochemical Stability of Real-Scale Metallic Nanoparticles. *Nat Commun* **2023**, *14* (1), 3004.

[R12] Sharma, R. K.; Jena, M. K.; Minhas, H.; Pathak, B. Machine-Learning-Assisted Screening of Nanocluster Electrocatalysts: Mapping and Reshaping the Activity Volcano for the Oxygen Reduction Reaction. *ACS Appl. Mater. Interfaces* **2024**, *16* (46), 63589–63601.

[R13] Qin, S.; Banerjee, S.; Sensoy, M. G.; Rappe, A. M. Unveiling the Electrocatalytic Hydrogen Evolution Reaction Pathway on RuP2 through Ab Initio Grand Canonical Monte Carlo. *ACS Catal.* **2024**, *14* (23), 17253–17262.

[R14] Zheng, S.; Zhang, X.-M.; Liu, H.-S.; Liang, G.-H.; Zhang, S.-W.; Zhang, W.; Wang, B.; Yang, J.; Jin, X.; Pan, F.; Li, J.-F. Active Phase Discovery in Heterogeneous Catalysis via Topology-Guided Sampling and Machine Learning. *Nat Commun* **2025**, *16* (1), 2542.

- The more complex LaMcO3 runs took 70 min on a 2x2 supercell. The manuscript would benefit a lot from using a larger supercell and inclusion of how the needed compute time scales with system size.

To address the scalability of VSSR-Pourbaix, we have performed additional $LaMnO_3(001)$ sampling experiments using **3×3 and 4×4 supercells**, which further demonstrate the method's ability to handle realistic, multi-adsorbate aqueous environments. These results are summarized in the SI as **Figs. S11 and S12**. We used the same sampling workflow as for the 2×2 system, focused on the bulk stability region, but with a **fine-tuned CHGNet model** instead of the pre-trained version. Notably, the **compute time scales sub-linearly** with the number of atoms:

- 2×2: 60 atoms → 70 min
- 3×3: 135 atoms → 140 min
- 4×4: 240 atoms → 210 min

These runtime values have been added to **Methods: Workflow management and compute time**. Updates have also been made to **Methods: Surface slab modeling**, **Methods: Surface analysis,** and the **Discussion** section to reflect the new experiments.

At $\boldsymbol{U_{SHE}}$ **= 0.6 V** and **pH = 12**, we obtained:

- **88 low-energy structures** for **3×3**
- **33 for 4×4**

Compared to **27 for 2×2**, all structures were selected based on their fine-tuned CHGNet ΔΩ values within 0.1 eV/surface unit cell (6.42 meV/$Å^2$) of the MnO_2-$\frac{1}{4}$OH* minimum.

We then constructed surface Pourbaix diagrams for **2×2, 3×3, 4×4**, and the **combined set** (Fig. S11(d-g)). With fine-tuned CHGNet, the following additional surface phases emerged:

- 2×2: 3 new phases
- 3×3: 7 new phases
- 4×4: 3 new phases

In the combined diagram (Fig. S11(g)), **2 of the 5 newly discovered Pourbaix domains** arose from a larger supercell, confirming their value in uncovering symmetry-breaking reconstructions not accessible via smaller supercells. We have added a paragraph to **Sampling additional surface phases** to highlight our new results.

Due to resource and time constraints, we have deferred **DFT-level energy evaluations and even larger supercell sampling** to future work. Nonetheless, these results demonstrate that VSSR-Pourbaix scales well with system size and can uncover diverse, low-energy reconstructions in **large, symmetry-breaking surface supercells**—structures that are unlikely to be found through manual, intuition-driven exploration alone.

- The long discussion and benchmark of pre-trained vs. fine-tuned foundational model does not really contribute to accelerated creation of pourbaix diagrams. Taking into account that the MACE-MP0 paper (https://arxiv.org/abs/2401.00096v2) already discourages taking the pre-trained model from scratch, it increases readability if the section (or at least major parts) Validation modified VSSR-MC and fine-tuning (pages 7-11) are shifted in the SI.

We thank the Reviewer for their feedback. Our main message is to demonstrate that pre-trained NFFs can already perform well on surfaces outside their training set *if* the chemical environments are sufficiently represented—such as in the case of $LaMnO_3(001)$. Conversely, when this is not the case—as with Pt(111)—our results show that additional sampling and fine-tuning with VSSR-MC still enables accurate predictions at significantly lower cost than training a model from scratch.

In addition, we highlight that VSSR-MC was able to recover all reference structures in the Pt(111) case and sample new low-energy phases in $LaMnO_3(001)$, especially near the bulk stability region.

We respectfully note that we do not interpret the MACE-MP0 paper [R15] as discouraging the use of pre-trained models as-is. While the authors acknowledge that pre-trained models may not yield quantitative accuracy for catalysis applications out-of-the-box, they also report robust out-of-distribution performance. In fact, their Pt(111) Pourbaix analysis showed good agreement with prior work, and they note that "its stability in MD and exploring reactive pathways is remarkable and provides a starting point for further optimisations."

That said, we agree with the Reviewer that our original section was overly long, which may have obscured our core message. We have therefore **restructured this section** to focus more clearly on the contrast between Pt and $LaMnO_3$ and **moved detailed benchmarking and fine-tuning results to the Methods section and the SI** to improve readability.

Reference:

[R15] Batatia, I.; Benner, P.; Chiang, Y.; Elena, A. M.; Kovács, D. P.; Riebesell, J.; Advincula, X. R.; Asta, M.; Avaylon, M.; Baldwin, W. J.; Berger, F.; Bernstein, N.; Bhowmik, A.; Blau, S. M.; Cărare, V.; Darby, J. P.; De, S.; Pia, F. D.; Deringer, V. L.; Elijošius, R.; El-Machachi, Z.; Falcioni, F.; Fako, E.; Ferrari, A. C.; Genreith-Schriever, A.; George, J.; Goodall, R. E. A.; Grey, C. P.; Grigorev, P.; Han, S.; Handley, W.; Heenen, H. H.; Hermansson, K.; Holm, C.; Jaafar, J.; Hofmann, S.; Jakob, K. S.; Jung, H.; Kapil, V.; Kaplan, A. D.; Karimitari, N.; Kermode, J. R.; Kroupa, N.; Kullgren, J.; Kuner, M. C.; Kuryla, D.; Liepuoniute, G.; Margraf, J. T.; Magdău, I.-B.; Michaelides, A.; Moore, J. H.; Naik, A. A.; Niblett, S. P.; Norwood, S. W.; O'Neill, N.; Ortner, C.; Persson, K. A.; Reuter, K.; Rosen, A. S.; Schaaf, L. L.; Schran, C.; Shi, B. X.; Sivonxay, E.; Stenczel, T. K.; Svahn, V.; Sutton, C.; Swinburne, T. D.; Tilly, J.; Oord, C. van der; Varga-Umbrich, E.; Vegge, T.; Vondrák, M.; Wang, Y.; Witt, W. C.; Zills, F.; Csányi, G. A Foundation Model for Atomistic Materials Chemistry. arXiv March 1, 2024. https://doi.org/10.48550/arXiv.2401.00096.

Minor comments:

- The calculations or sources from which the $\Delta\Omega_{\mathrm{surf}}$ values originate are missing. A table with the values used for $\Delta\Omega_{\mathrm{surf}}$ and μ_{A} would greatly benefit reproducibility.

We believe the Reviewer is requesting for $\Delta\Omega^{\ominus}_{\mathrm{A,SHE}}$, the standard-state free energy of reaction for each species A, and $\Delta\Omega^{\ominus}_{\mathrm{A,SHE}} - 2.3 N_{\mathrm{A,H^+}} k_{\mathrm{B}} T \mathrm{pH} - N_{\mathrm{A,e}}(eU_{\mathrm{SHE}}) + k_{\mathrm{B}} T \ln a_{\mathrm{H_xAO_y^{z-}}}$, the condition-dependent free energy of reaction for each species A. While we obtained these values algorithmically from the Materials Project (See **Methods: Species Pourbaix diagram construction** and **Methods: Surface Pourbaix grand potential**) and included them in our **Zenodo dataset**, we provide the tables for Pt and $LaMnO_3$ as requested here (**Tables R3 & R4**) and in the SI (**Tables S1 & S2**). We also point readers to these tables and our Zenodo dataset in

Methods: Surface Pourbaix grand potential. μ_A in **Eqn. 5** is equivalent to $\mu_A^{\ominus}$ and has been updated. Additionally, we had typos near **Eqn. 3** and in **Eqn. 5**, which have now been corrected.

A	$H_xAO_y^{Z-}$	$\Delta\Omega_{A,SHE}^{\ominus}$ /eV	$\Delta\Omega_{A,SHE}^{\ominus} - 2.3N_{A,H^+}k_BT\text{pH} - N_{A,e}(eU_{SHE}) + k_BT\ln a_{H_xAO_y^{z-}}$/eV
Pt	Pt(s)	0	0
	Pt_3O_4(s)	1.771	$1.771 - 0.158\text{pH} - 2.667eU_{SHE}$
	PtO_2(s)	2.770	$2.770 - 0.236\text{pH} - 4eU_{SHE}$
O	H_2O	−2.458	$-2.458 + 0.118\text{pH} + 2eU_{SHE}$
H	H^+	0	$-0.0591\text{pH} - eU_{SHE}$

Table R3. Free energy of reaction for each Pt species at 298 K.

A	$H_xAO_y^{Z-}$	$\Delta\Omega_{A,SHE}^{\ominus}$ /eV	$\Delta\Omega_{A,SHE}^{\ominus} - 2.3N_{A,H^+}k_BT\text{pH} - N_{A,e}(eU_{SHE}) + k_BT\ln a_{H_xAO_y^{z-}}$/eV
Mn	Mn^{2+}	−2.316	$-2.316 - 2eU_{SHE} + 0.0257\ln a_{Mn^{2+}}$
	MnO(s)	−1.183	$-1.183 - 0.118\text{pH} - 2eU_{SHE}$
	MnO_2(s)	0.133	$0.133 - 0.236\text{pH} - 4eU_{SHE}$
	Mn_2O_3(s)	−0.872	$-0.872 - 0.177\text{pH} - 3eU_{SHE}$
	Mn_3O_4(s)	−1.084	$-1.084 - 0.158\text{pH} - 2.667eU_{SHE}$
	MnO_4^-	5.246	$5.246 - 0.472\text{pH} - 7eU_{SHE} + 0.0257\ln a_{MnO_4^-}$
	MnO_4^{2-}	4.691	$4.691 - 0.472\text{pH} - 6eU_{SHE} + 0.0257\ln a_{MnO_4^{2-}}$
	$MnOH^+$	−1.694	$-1.694 - 0.0591\text{pH} - 2eU_{SHE} + 0.0257\ln a_{MnOH^+}$
	$Mn(OH)_3^-$	−0.293	$-0.293 - 0.177\text{pH} - 2eU_{SHE} + 0.0257\ln a_{Mn(OH)_3^-}$
La	La^{3+}	−7.450	$-7.450 - 3eU_{SHE} + 0.0257\ln a_{La^{3+}}$
	$LaHO_2$(s)	−5.593	$-5.593 - 0.177\text{pH} - 3eU_{SHE}$
O	H_2O	−2.458	$-2.458 + 0.118\text{pH} + 2eU_{SHE}$
H	H^+	0	$-0.0591\text{pH} - eU_{SHE}$

Table R4. Free energy of reaction for each $LaMnO_3$ species at 298 K.

- The definition of a sweep is not completely clear.

A sweep is an implementation-specific way of tracking/grouping MC iterations. In this work, we defined one sweep to consist of 20 MC sampling iterations. We have removed the usage of "sweep" from the main text to avoid confusion.

- The VSSR-MC is performed semigrand-canonical, which puts limitations on the covered composition space: Can you state how you cover the necessary composition space for your finite-sized cell?

We are semigrand-canonical in the sense that the total number of virtual sites—including both filled and empty sites—is fixed, while both the **elemental identities** of the filled sites and the **number of filled vs. empty sites** are allowed to vary. In principle, the number of accessible compositions is limited by the number of virtual sites. However, by allocating at least as many virtual surface sites as atoms in the pristine surface layer and including the pristine surface atoms in our virtual sites (as done in this work), we enable sampling over a broad compositional space. For perovskite oxide systems, this setup allowed us to **sample O(100) unique compositions**, **substantially exceeding the O(1–10) typically explored in prior approaches** that either rely on hand-picked compositions or perform limited sampling. (See Tables R1 & R2.) In both benchmark cases, our method not only **recovered known terminations** but also **revealed low-energy surface phases previously unreported in the literature**.

- The geometry optimizations are limited to 20 steps (page 20). Why is the step limit set so low? How often was that limit reached?

During VSSR-Pourbaix sampling, we set a 20-step limit on relaxations as a heuristic to reduce computational cost and to prevent atoms from drifting too far from their assigned virtual surface sites. Our inductive bias is that, since virtual surface sites are initialized close to reasonable positions, only a small number of steps is typically required to explore the relevant configurational space. In practice, the 20-step limit was reached in about half of the cases. When it was, the average residual forces were generally below 0.5 eV/Å. Importantly, we do not aim for full convergence at this stage; instead, we prefer non-zero forces to provide informative gradients for NFF fine-tuning. For downstream Pourbaix analysis, low-energy structures predicted by the fine-tuned NFF were subsequently relaxed fully at the DFT level to ensure accurate surface stability comparisons.

- Why is the surface grand potential normalized by the unit cell and not the surface area as commonly done in ab-initio thermodynamics?

Thank you for pointing this out. We have revised our analysis to normalize the surface Pourbaix grand potential by surface area (eV/Å^2), in accordance with standard *ab initio* thermodynamic conventions. All affected figures and corresponding text have been

updated. Where prior cutoff criteria were expressed in eV per surface unit cell, we now also report the surface-area normalized values in parentheses for clarity.

oc-2025-00547m.R2

Name: Peer Review Information for "Accelerating and enhancing thermodynamic simulations of electrochemical interfaces"

Second Round of Reviewer Comments

Reviewer: 2

Comments to the Author

All comments were sufficently addressed, leading to high quality science and a very good manuscript that is suitable for publication in ACS central science.

Reviewer: 1

Comments to the Author

The authors have addressed all comments that I raised in my initial evaluation of their manuscript. Therefore, I recommend this article for publication in ACS Central Science.

I have one final remark concerning the response to point D): The authors said that they screened the literature for a reference Pourbaix diagram of Pt(111). Here, the authors may take a look at the following article, which appears to be an extension of the reference R2-R3 listed in the authors' response:

https://www.nature.com/articles/s41929-020-0497-y

If I remember the discussion of the theoretical part correctly, the authors of this article discuss surface reconstruction of the Pt(111) surface.

I suppose that surface reconstruction is not part of the authors' approach and the constructed Pourbaix diagrams.

Therefore, I would appreciate if this could be shorty discussed in the manuscript in how far surface reconstructions, such as the one reported for Pt(111) by Magnussen et al are considered in the authors' method or could be incorporated into the framework.

Author's Response to Peer Review Comments:

We thank the Editor and Reviewers for the acceptance. The final Response to Reviewers is attached.

oc-2025-00547m.R1: Accelerating and enhancing thermodynamic simulations of electrochemical interfaces

Reviewer: 1

Recommendation: Publish in ACS Central Science after minor revisions noted.

Comments:
The authors have addressed all comments that I raised in my initial evaluation of their manuscript. Therefore, I recommend this article for publication in ACS Central Science.
I have one final remark concerning the response to point D): The authors said that they screened the literature for a reference Pourbaix diagram of Pt(111). Here, the authors may take a look at the following article, which appears to be an extension of the reference R2-R3 listed in the authors' response: https://www.nature.com/articles/s41929-020-0497-y
If I remember the discussion of the theoretical part correctly, the authors of this article discuss surface reconstruction of the Pt(111) surface.
I suppose that surface reconstruction is not part of the authors' approach and the constructed Pourbaix diagrams.
Therefore, I would appreciate if this could be shorty discussed in the manuscript in how far surface reconstructions, such as the one reported for Pt(111) by Magnussen et al are considered in the authors' method or could be incorporated into the framework.

We thank the Reviewer for their recommendation and thoughtful final remark. In the referenced work [R1] and related studies [R2–R3], Magnussen and co-workers report place-exchange (PE) reconstructions on Pt(111), where a surface Pt atom is displaced from its lattice site and the vacancy is often filled by an oxygen atom. We did not observe such PE structures among the low-energy configurations sampled in our work. This may reflect a limitation of using a pre-trained NFF during the initial sampling stage. Performing a second round of VSSR-MC after fine-tuning the NFF could enable exploration of a broader configurational space, potentially capturing PE-like reconstructions and associated relaxations. We have added a brief discussion of this point to **Sampling additional surface phases** in the manuscript.

References:
[R1] Fuchs, T.; Drnec, J.; Calle-Vallejo, F.; Stubb, N.; Sandbeck, D. J. S.; Ruge, M.; Cherevko, S.; Harrington, D. A.; Magnussen, O. M. Structure Dependency of the Atomic-Scale Mechanisms of Platinum Electro-Oxidation and Dissolution. *Nat Catal* **2020**, *3* (9), 754–761. https://doi.org/10.1038/s41929-020-0497-y.

[R2] Ruge, M.; Drnec, J.; Rahn, B.; Reikowski, F.; Harrington, D. A.; Carlà, F.; Felici, R.; Stettner, J.; Magnussen, O. M. Structural Reorganization of Pt(111) Electrodes by Electrochemical Oxidation and Reduction. *J. Am. Chem. Soc.* **2017**, *139* (12), 4532–4539. https://doi.org/10.1021/jacs.7b01039.

[R3] Drnec, J.; Ruge, M.; Reikowski, F.; Rahn, B.; Carlà, F.; Felici, R.; Stettner, J.; Magnussen, O. M.; Harrington, D. A. Initial Stages of Pt(111) Electrooxidation: Dynamic and Structural Studies by Surface X-Ray Diffraction. *Electrochimica Acta* **2017**, *224*, 220–227. https://doi.org/10.1016/j.electacta.2016.12.028.

oc-2025-00547m.R1: Accelerating and enhancing thermodynamic simulations of electrochemical interfaces

Reviewer: 2

Recommendation: Publish in ACS Central Science without change.

Comments:
All comments were sufficently addressed, leading to high quality science and a very good manuscript that is suitable for publication in ACS central science.

Additional Questions:
Quality of experimental data, technical rigor: Top 1%

Significance to chemistry researchers in this and related fields: Top 1%

Broad interest to other researchers: Top 10%

Novelty: Top 1%

Is this research study suitable for media coverage or a First Reactions (a News & Views piece in the journal)?: No

We sincerely thank the Reviewer for their supportive comments.
